# Evaluation of Approximate Fourth-Order N‑Electron Valence Perturbation Theory (NEVPT4(SD)) for the Excited States of Organic Molecules

**DOI:** 10.1021/acs.jctc.5c01685

**Published:** 2025-11-20

**Authors:** Emily M. Kempfer, Kantharuban Sivalingam, Frank Neese

**Affiliations:** 28314 Max Planck Institut für Kohlenforschung, Kaiser-Wilhelm Platz 1, Mülheim an der Ruhr D-45472, Germany

## Abstract

In this work, we assess the accuracy of the approximate fourth-order N-electron valence perturbation theory (NEVPT4­(SD)) methodology for computing excited states of organic molecules. The well-established Thiel benchmark set was employed, comprising 225 vertical excitations spanning π → π*, *n* → π*, and σ → π* transition types. A state-specific canonicalization procedure was applied, enabling a direct comparison with CC3 reference data reported by Schreiber et al. *J. Chem. Phys.*, **2008**, *128*, 134110. For both singlet and triplet excitations, NEVPT4­(SD) systematically outperforms lower-order NEVPT variants, as well as previously reported complete active space second-order perturbation theory (CASPT2) results. A detailed analysis of the singlet excitations reveals that *n* → π* transitions have a slight tendency to be overestimated (by about 0.1 eV), while π → π* excitations tend to be slightly underestimated (by −0.04 eV). While this shift persists across all NEVPT perturbation orders, its magnitude decreases with higher-order treatments. Across the entire test set, NEVPT4­(SD) has a very narrow error distribution with a peak very close to 0. Thus, this study demonstrates the robustness and high accuracy of NEVPT4­(SD) for vertical excitation energies, highlighting its clear advantages over lower-order perturbative approaches while remaining computationally much more affordable than other multireference correlation approaches that proceed beyond second-order perturbation theory.

## Graphical abstract

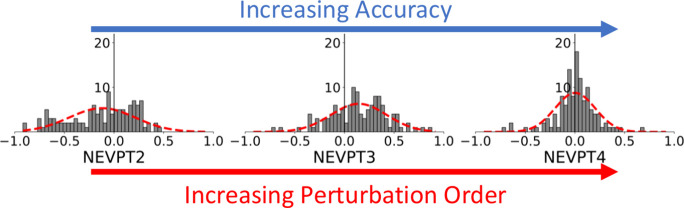

## 1 Introduction

Benchmark sets have long played a crucial role in assessing the accuracy of computational methods in electronic structure theory. Among these, the Thiel benchmark set stands out as one of the most widely used for evaluating vertical excitation energies (VEEs) of small- to medium-sized closed-shell organic molecules.^1^ This set comprises 223 VEEs across 28 molecules, covering key chromophore classes, including π → π*, *n* → π*, and σ → π* excitations. In the original study, Schreiber et al.^1^ established high-accuracy reference values using rigorous ab initio methods such as complete active space second-order perturbation theory (CASPT2)^2,3^ and a hierarchy of coupled-cluster linear response (CC-LR) approaches, including CC2, CCSD, and CC3.^4–9^ These methods remain benchmarks for excited-state calculations due to their reliability and systematic convergence. The CC3 methodology, in particular, has long been recognized as a reliable and accurate single-reference method for these calculations. Numerous studies have demonstrated its excellent agreement with full configuration interaction (FCI) results in systems where a single-reference description is appropriate,^6,7,10–16^ making it a widely accepted benchmark for evaluating the performance of more approximate methods.

Since its introduction, the Thiel benchmark set has been widely adopted to evaluate the accuracy of a broad spectrum of excited-state electronic structure methods, extending well beyond those originally considered. Studies have leveraged this set to assess the performance of numerous computational approaches systematically. Among these, density functional theory (DFT) has been explored with a range of exchange–correlation functionals, including conventional hybrids such as BP86, B3LYP, and BHLYP,^17^ as well as more sophisticated forms like local hybrid functionals^18^ and projected double-hybrid functionals.^19^ Extensions of the DFT framework, such as the semiempirical DFT-based multireference configuration interaction (DFT/MRCI) method, have also been benchmarked.^17^ In addition, single-reference wave function-based methods, including the variationally optimized configuration interaction single (VOA-CIS) approach developed by Liu and Subotnik^20^ and various flavors of algebraic diagrammatic construction (ADC),^21,22^ have been evaluated. Multireference methods, such as the second-order N-electron valence state perturbation theory (NEVPT2), have also been investigated using this benchmark.^23^

Among the multireference perturbative approaches, NEVPT2 has emerged as a particularly robust and reliable method, offering several key advantages over the more widely known complete active space second-order perturbation theory (CASPT2). Notably, NEVPT2 is fully intruder state free by construction and benefits from a strictly size-consistent formulation,^24–26^ ensuring more stable and physically meaningful results across diverse chemical systems.^27–30^ For a comprehensive and detailed overview of the NEVPT2 methodology, we refer the reader to the seminal work by Angeli et al. in refs 24–26 ,^31^, and^32^, which provides a comprehensive foundation for understanding both the theoretical framework and its practical application.

There have also been important extensions to the NEVPT2 methodology including but not limited to domain-based local pair natural orbitals NEVPT2 (DLPNO-NEVPT2),^33^ F12-NEVPT2,^34^ and ICE-NEVPT2,^35^ strongly and partially contracted NEVPT2 (SC-NEVPT2 and PC-NEVPT2),^36^ time-dependent (*t*-)­NEVPT2,^37^ and NEVPTS.^38^ Although NEVPT2 and related second-order perturbation methods have been widely investigated, their accuracy remains fundamentally limited by the order of the perturbation theory they employ. Previous studies of both single and multireference methodologies have demonstrated significantly greater accuracy when increasing to fourth-order corrections.^39,40^ Although third-order corrections have been advocated in certain multireference contexts, they often present challenges in both single- and multireference formulations.^40^ Our previous findings demonstrated that third-order approaches can, in some cases, yield less reliable results than either second- or fourth-order treatments.^41^ Thus, a key motivation for this study is to further demonstrate the accuracy of the fourth-order perturbed NEVPT methodology, specifically utilizing VEEs. This methodology has been previously termed as NEVPT4­(SD),^41^ denoting that it is the fully internally contracted variant^42^ of the fourth-order perturbed NEVPT with the excitation space restricted to the first-order interacting space (FOIS), consisting of internally contracted single and double excitations from the CASSCF reference space. It was pointed out by Grimme et al.^40^ that in order for the method to remain size consistent while neglecting quadruple excitations, it is necessary to drop the fourth-order renormalization terms, and consequently, this is done in the NEVPT4­(SD) method.^41^ Building on the work of Schapiro et al.,^23^ this study applies the newly developed FIC-NEVPT4­(SD) approach for the calculation of vertical excitation energies (VEEs). Angeli and co-workers have previously demonstrated some challenging cases of VEEs using the FIC-NEVPT2 methodology, specifically ethene,^43,44^ all-*E*-octatetraene,^45^ furan,^46^ pyrrole,^47,48^ *s*-tetrazine,^49^ formaldehyde,^50,51^ and acetone.^43–51^ They mitigated some of these issues through the use of enlarged active spaces and diffuse basis sets. Here, we assess whether FIC-NEVPT4­(SD) can improve upon these results while maintaining consistency with prior studies in terms of active space selection, state averaging, and basis sets.^1^ This approach ensures direct comparability with established benchmarks, specifically CC3.

## 2 Computational Details

The Thiel benchmark set was extensively utilized in prior theoretical studies. In this work, we employ the optimized molecular geometries reported by Schreiber et al.^1^ and Schapiro et al.,^23^ obtained at the MP2/6-31G* level of theory, and adopt the TZVP basis set for all subsequent calculations. Although the ORCA framework supports the computation of state-averaged energies across different spatial and spin symmetries, we chose to perform these calculations separately to allow for direct comparison with previous work.^1^ State-averaged CASSCF is employed when multiple roots of the same symmetry are required. VEEs are computed by using two distinct protocols, depending on the symmetry and number of roots involved. For excitations within the same irreducible representation as the ground state, the ground state energy is obtained from the state-averaged calculation and used as the zeroth-order reference. In contrast, when the excited state differs in symmetry from the ground state, the state-specific energy of the ground state is used as the zeroth-order reference. For a more complete understanding of the computation of the VEEs, we refer the reader to the Supporting Information. We also set the orbital gradient convergence and CASSCF energy thresholds to 10^–5^ and 10^–9^ hartree, respectively, for obtaining the most accurate comparison with previous published results. It is also noted that if an *n* → π* excitation is included, then the nonbonding orbitals are included in the CASSCF orbital optimization.

The FIC-NEVPT4­(SD) calculations were carried out using a development version of ORCA based on ORCA 6.1.^52^ Our previous publication has detailed the implementation of the FIC-NEVPT4­(SD) methodology, which utilizes the ORCA-AGE (Automatic Generator for Electronic structure methods) toolchain.^53^ This toolchain, described comprehensively elsewhere,^41^ builds upon the existing FIC-NEVPT2 implementation in ORCA-AGE, which serves as an initial guess for the FIC-MRCI method.^42^

A previous study conducted by our group has also examined the influence of orbital canonicalization on FIC-NEVPT2 vertical excitation energies.^23^ Two distinct procedures can be employed to achieve orbital canonicalization:

(1) The more accurate state-specific approach, in which orbital energies and orbitals are obtained by diagonalizing the state-specific Fock operator (called within orca as canonstep = canonical), and
(2) the approximate approach, where orbital energies are taken as the diagonal elements of the state-specific Fock operator, while the orbitals remain state-averaged and pseudocanonicalized (canonstep = 0).

The original work detailing the FIC-NEVPT4­(SD) methodology specifically utilizes the second option or the “approximate approach.”^41^ Earlier calculations demonstrated that the choice of the canonicalization procedure had a negligible impact on the test set, particularly within the FIC-NEVPT2 framework.^23^ In the present work, we performed all calculations using both canonicalization schemes and found that this choice similarly has a minimal effect on FIC-NEVPT4­(SD) results. Thus, unless otherwise noted, all data presented in the preceding sections were obtained using the state-specific Fock operator (option 1). Results obtained with the approximate canonicalization scheme (option 2) are provided in the Supporting Information.

## 3 Results and Discussion

This section is structured into three parts. First, we present the results from the singlet and triplet excitation energies across the full Thiel benchmark set. Second, we focus on singlet excitations, presenting a comprehensive statistical analysis and breaking down the types of singlet excitations by excitation classes. Finally, we examine triplet excitations, comparing their behavior to that of singlet counterparts and discussing methodological implications. For conciseness, the “FIC-” prefix is omitted in the subsequent discussion. All methods implicitly employ the FIC- formalism, unless stated otherwise.

### 3.1 Benchmarking Set

We present benchmark results for 153 singlet and 72 triplet vertical excitation energies (VEEs) computed by using NEVPT methods, with CC3 serving as the reference standard (Tables 1 and 2). The excited states considered in this work follow the selection by Schapiro et al.,^23^ who included two additional excitations (the singlet and triplet B_3g_ states of *s*-tetrazine) not present in the original Thiel benchmark set. However, these states exhibit significant double excitation character and, consequently, lack direct CC3 reference values.

**Table 1:** Singlet Vertical Excitation Energies Δ*E* (eV) (Canonicalization Is State Specific)

molecule	state	type	NEVPT2	NEVPT3	NEVPT4	CC3
ethene	1 ^1^B_1u_	π → π*	8.64	8.74	8.45	8.37
*E*-butadiene	1 ^1^B_u_	π → π*	6.21	6.93	6.57	6.58
	2 ^1^A_g_	π → π*	6.80	6.56	6.63	6.77
all-*E*-hexatriene	1 ^1^B_u_	π → π*	4.84	5.94	5.53	5.58
	2 ^1^A_g_	π → π*	5.56	5.44	5.47	5.72
all-*E*-octatetraene	2 ^1^A_g_	π → π*	4.72	4.64	4.68	4.97
	1 ^1^B_u_	π → π*	4.04	5.30	4.87	4.94
	2 ^1^B_u_	π → π*	5.86	5.85	5.87	6.06
	3 ^1^A_g_	π → π*	5.97	7.13	6.66	6.50
	4 ^1^A_g_	π → π*	6.67	6.46	6.51	6.81
	3 ^1^B_u_	π → π*	8.35	8.20	8.25	7.91
cyclopropene	1 ^1^B_1_	σ → π*	6.84	6.95	6.91	6.90
	1 ^1^B_2_	π → π*	7.03	7.40	7.14	7.10
cyclopentadiene	1 ^1^B_2_	π → π*	5.21	6.05	5.67	5.73
	2 ^1^A_1_	π → π*	6.72	6.53	6.56	6.61
	3 ^1^A_1_	π → π*	8.22	9.05	8.55	8.69
norbornadiene	1 ^1^A_2_	π → π*	5.04	6.02	5.64	5.64
	1 ^1^B_2_	π → π*	5.80	7.02	6.44	6.49
	2 ^1^B_2_	π → π*	6.98	8.16	7.58	7.64
	2 ^1^A_2_	π → π*	7.04	8.20	7.69	7.71
benzene	1 ^1^B_2u_	π → π*	5.23	5.00	5.06	5.07
	1 ^1^B_1u_	π → π*	6.40	6.82	6.66	6.68
	1 ^1^E_1u_	π → π*	7.11	7.75	7.39	7.45
	2 ^1^E_2g_	π → π*	8.42	8.17	8.24	8.43
naphthalene	1 ^1^B_3u_	π → π*	4.37	4.25	4.30	4.27
	1 ^1^B_2u_	π → π*	4.37	5.27	4.99	5.03
	2 ^1^A_g_	π → π*	6.23	5.86	5.98	5.98
	1 ^1^B_1g_	π → π*	6.10	6.18	6.17	6.07
	2 ^1^B_3u_	π → π*	5.61	6.68	6.25	6.33
	2 ^1^B_1g_	π → π*	6.17	7.03	6.73	6.79
	2 ^1^B_2u_	π → π*	6.01	6.81	6.53	6.57
	3 ^1^A_g_	π → π*	6.85	6.70	6.77	6.90
	3 ^1^B_2u_	π → π*	7.81	8.70	8.31	8.44
	3 ^1^B_3u_	π → π*	7.92	7.78	7.84	8.12
furan	1 ^1^B_2_	π → π*	6.44	7.02	6.72	6.60
	2 ^1^A_1_	π → π*	6.75	6.61	6.65	6.62
	3 ^1^A_1_	π → π*	8.35	8.96	8.59	8.53
pyrrole	2 ^1^A_1_	π → π*	6.56	6.39	6.44	6.40
	1 ^1^B_2_	π → π*	6.78	7.10	6.92	6.71
	3 ^1^A_1_	π → π*	8.19	8.53	8.29	8.17
imidazole	1 ^1^A″	*n* → π*	6.97	6.80	6.83	6.82
	2 ^1^A′	π → π*	6.80	6.71	6.72	6.58
	3 ^1^A′	π → π*	6.85	7.21	7.03	7.10
	2 ^1^A″	*n* → π*	8.01	7.97	7.97	7.93
	4 ^1^A′	π → π*	8.39	8.78	8.48	8.45
pyridine	1 ^1^B_2_	π → π*	5.33	5.10	5.18	5.15
	1 ^1^B_1_	*n* → π*	5.26	5.14	5.14	5.05
	1 ^1^A_2_	*n* → π*	5.46	5.62	5.53	5.50
	2 ^1^A_1_	π → π*	7.09	7.33	7.28	6.85
	3 ^1^A_1_	π → π*	7.23	8.05	7.62	7.70
	2 ^1^B_2_	π → π*	7.10	8.09	7.60	7.59
	4 ^1^A_1_	π → π*	8.03	8.05	8.06	8.68
	3 ^1^B_2_	π → π*	8.53	8.30	8.35	8.77
pyrazine	1 ^1^B_3u_	*n* → π*	4.20	4.36	4.34	4.24
	1 ^1^A_u_	*n* → π*	4.93	5.27	5.19	5.05
	1 ^1^B_2u_	π → π*	5.31	5.03	5.08	5.02
	1 ^1^B_2g_	*n* → π*	5.86	5.68	5.75	5.74
	1 ^1^B_1g_	*n* → π*	6.77	6.86	6.87	6.75
	1 ^1^B_1u_	π → π*	6.76	7.25	7.04	7.07
	2 ^1^B_1u_	π → π*	7.72	8.53	7.98	8.06
	2 ^1^B_2u_	π → π*	7.43	8.31	7.87	8.05
	1 ^1^B_3g_	π → π*	8.73	8.46	8.55	8.77
	2 ^1^A_g_	π → π*	8.87	8.51	8.61	8.69
pyrimidine	1 ^1^B_1_	*n* → π*	4.52	4.58	4.57	4.50
	1 ^1^A_2_	*n* → π*	4.81	5.02	4.98	4.93
	1 ^1^B_2_	π → π*	5.61	5.32	5.39	5.36
	2 ^1^A_1_	π → π*	7.42	7.58	7.50	7.06
	2 ^1^B_2_	π → π*	7.51	8.44	7.93	8.01
	3 ^1^A_1_	π → π*	7.74	8.46	8.06	7.74
pyridazine	1 ^1^B_1_	*n* → π*	3.92	4.08	4.03	3.92
	1 ^1^A_2_	*n* → π*	4.57	4.67	4.65	4.49
	2 ^1^A_1_	π → π*	5.46	5.12	5.24	5.22
	2 ^1^A_2_	*n* → π*	5.89	5.87	5.86	5.74
	2 ^1^B_1_	*n* → π*	6.68	6.68	6.65	6.41
	1 ^1^B_2_	π → π*	7.34	7.78	7.60	6.93
	2 ^1^B_2_	π → π*	7.25	8.08	7.61	7.55
	3 ^1^A_1_	π → π*	7.38	8.32	7.77	7.82
*s*-triazine	1 ^1^A_1_″	*n* → π*	4.65	5.08	4.99	4.78
	1 ^1^A_2_″	*n* → π*	4.88	4.85	4.90	4.76
	1 ^1^E″	*n* → π*	4.87	5.01	5.01	4.81
	1 ^1^A_2_′	π → π*	5.92	5.63	5.75	5.71
	2 ^1^A_1_′	π → π*	7.20	7.71	7.51	7.41
	2 ^1^E″	*n* → π*	7.98	7.89	7.93	7.80
	1 ^1^E′	π → π*	7.94	8.81	8.35	8.04
	2 ^1^E′	π → π*	9.01	8.73	8.80	9.44
*s*-tetrazine	1 ^1^B_3u_	*n* → π*	2.41	2.63	2.61	2.53
	1 ^1^A_u_	π → π*	3.76	3.97	3.97	3.79
	1 ^1^B_1g_	*n* → π*	5.17	5.04	5.12	4.97
	1 ^1^B_2u_	π → π*	5.47	4.98	5.17	5.12
	1 ^1^B_2g_	*n* → π*	5.53	5.20	5.35	5.34
	1 ^1^B_3g_	*n*,*n* → π*,π*	6.30	6.37	6.40	
	2 ^1^A_u_	π → π*	5.70	5.69	5.69	5.46
	2 ^1^B_2g_	*n* → π*	6.28	6.30	6.36	6.23
	2 ^1^B_1g_	*n* → π*	6.83	6.70	6.82	6.87
	3 ^1^B_1g_	*n* → π*	7.02	6.94	7.04	7.08
	2 ^1^B_3u_	*n* → π*	7.11	7.03	7.06	6.67
	1 ^1^B_1u_	π → π*	6.76	7.56	7.31	7.45
	2 ^1^B_1u_	π → π*	6.87	7.97	7.40	7.79
	2 ^1^B_2u_	π → π*	8.33	8.16	8.22	8.51
	2 ^1^B_3g_	π → π*	8.10	8.51	8.37	8.47
formaldehyde	1 ^1^A_2_	*n* → π*	4.22	4.00	4.02	3.95
	1 ^1^B_1_	σ → π*	9.40	9.21	9.23	9.18
	2 ^1^A_1_	π → π*	8.79	10.18	9.20	9.53
acetone	1 ^1^A_2_	*n* → π*	4.47	4.54	4.47	4.40
	1 ^1^B_1_	σ → π*	9.50	9.55	9.42	9.17
	2 ^1^A_1_	π → π*	9.28	9.48	9.17	9.65
*p*-benzoquinone	1 ^1^A_u_	*n* → π*	2.99	3.08	3.05	2.85
	1 ^1^B_1g_	*n* → π*	3.00	3.07	3.03	2.75
	1 ^1^B_3g_	π → π*	4.35	4.97	4.83	4.59
	1 ^1^B_1u_	π → π*	4.85	6.01	5.61	5.62
	1 ^1^B_3u_	*n* → π*	5.88	5.95	5.94	5.82
	2 ^1^B_3g_	π → π*	6.70	7.35	7.22	7.27
	2 ^1^B_1u_	π → π*	7.72	7.80	7.83	7.82
formamide	1 ^1^A″	*n* → π*	5.93	5.54	5.65	5.65
	2 ^1^A′	π → π*	7.58	8.01	7.57	8.27
	3 ^1^A′	π → π*	10.75	11.07	10.74	10.93
acetamide	1 ^1^A″	*n* → π*	5.97	5.60	5.69	5.69
	2 ^1^A′	π → π*	7.48	7.96	7.53	7.67
	3 ^1^A′	π → π*	10.28	10.76	10.38	10.50
propanamide	1 ^1^A″	*n* → π*	5.98	5.62	5.73	5.72
	2 ^1^A′	π → π*	7.42	7.93	7.48	7.62
	3 ^1^A′	π → π*	10.14	10.64	10.23	10.06
cytosine	2 ^1^A′	π → π*	4.70	4.80	4.74	4.72^a^
	1 ^1^A″	*n* → π*	5.50	5.29	5.38	5.16^a^
	2 ^1^A″	*n* → π*	5.73	5.57	5.65	5.52^a^
	3 ^1^A′	π → π*	5.65	5.87	5.78	5.61^a^
	4 ^1^A′	π → π*	6.47	6.91	6.66	6.61^a^
	5 ^1^A′	π → π*	6.83	7.17	7.02	
	6 ^1^A′	π → π*	8.06	8.26	8.16	
thymine	1 ^1^A″	*n* → π*	4.96	4.97	4.98	4.98^a^
	2 ^1^A′	π → π*	5.05	5.55	5.33	5.34^a^
	3 ^1^A′	π → π*	6.34	7.00	6.66	6.34^a^
	2 ^1^A″	*n* → π*	6.49	6.47	6.49	6.45^a^
	4 ^1^A′	π → π*	6.43	6.63	6.51	6.71^a^
	3 ^1^A″	*n* → π*	6.69	7.16	7.00	
	4 ^1^A″	*n* → π*	7.41	7.64	7.59	
	5 ^1^A′	π → π*	7.36	7.90	7.56	
uracil	1 ^1^A″	*n* → π*	4.92	4.92	4.94	4.90^a^
	2 ^1^A′	π → π*	5.27	5.63	5.48	5.44^a^
	3 ^1^A′	π → π*	6.22	6.54	6.40	6.29^a^
	2 ^1^A″	*n* → π*	6.42	6.40	6.42	6.32^a^
	3 ^1^A″	*n* → π*	6.70	7.17	7.01	6.77^a^
	4 ^1^A′	π → π*	6.68	7.09	6.86	6.84^a^
	4 ^1^A″	*n* → π*	7.27	7.49	7.43	7.12^a^
	5 ^1^A′	π → π*	7.38	7.83	7.54	7.93^a^
adenine	2 ^1^A′	π → π*	5.08	5.61	5.42	5.18^a^
	3 ^1^A′	π → π*	5.42	5.23	5.30	5.39^a^
	1 ^1^A″	*n* → π*	5.35	5.47	5.47	5.34^a^
	2 ^1^A″	*n* → π*	6.07	6.12	6.13	5.96^a^
	4 ^1^A′	π → π*	6.48	7.04	6.80	6.53^a^
	5 ^1^A′	π → π*	6.82	7.03	6.93	
	6 ^1^A′	π → π*	6.92	6.83	6.84	
	7 ^1^A′	π → π*	7.69	7.85	7.79	

a The value is taken from ref 54.

**Table 2:** Triplet Vertical Excitation Energies Δ*E* (eV) (Canonicalization Is State Specific)

molecule	state	type	NEVPT2	NEVPT3	NEVPT4	CC3
ethene	1 ^3^B_1u_	π → π*	4.60	4.43	4.47	4.48
*E*-butadiene	1 ^3^B_u_	π → π*	3.38	3.33	3.36	3.32
	1 ^3^A_g_	π → π*	5.27	5.13	5.18	5.17
all-*E*-hexatriene	1 ^3^B_u_	π → π*	2.73	2.72	2.74	2.69
	1 ^3^A_g_	π → π*	4.39	4.31	4.35	4.32
all-*E*-octatetraene	1 ^3^B_u_	π → π*	2.32	2.34	2.36	2.30
	1 ^3^A_g_	π → π*	3.72	3.68	3.72	3.67
cyclopropene	1 ^3^B_2_	π → π*	4.50	4.32	4.35	4.34
	1 ^3^B_1_	σ → π*	6.57	6.64	6.63	6.62
cyclopentadiene	1 ^3^B_2_	π → π*	3.32	3.25	3.28	3.25
	1 ^3^A_1_	π → π*	5.22	5.06	5.11	5.09
norbornadine	1 ^3^A_2_	π → π*	3.80	3.78	3.80	3.72
	1 ^3^B_2_	π → π*	4.30	4.20	4.22	4.16
benzene	1 ^3^B_1u_	π → π*	4.31	4.06	4.14	4.12
	1 ^3^E_1u_	π → π*	4.98	4.90	4.92	4.90
	1 ^3^B_2u_	π → π*	5.47	6.19	5.97	6.04
	1 ^3^E_2g_	π → π*	7.59	7.31	7.40	7.49
naphthalene	1 ^3^B_2u_	π → π*	3.26	3.12	3.18	3.11
	1 ^3^B_3u_	π → π*	4.24	4.20	4.23	4.18
	1 ^3^B_1g_	π → π*	4.57	4.46	4.51	4.47
	2 ^3^B_2u_	π → π*	4.70	4.63	4.67	4.64
	2 ^3^B_3u_	π → π*	4.44	5.28	5.05	5.11
	1 ^3^A_g_	π → π*	5.59	5.52	5.57	5.52
	2 ^3^B_1g_	π → π*	5.80	6.78	6.45	6.48
	2 ^3^A_g_	π → π*	6.11	6.97	6.73	6.47
	3 ^3^A_g_	π → π*	6.52	6.37	6.43	6.79
	3 ^3^B_1g_	π → π*	6.79	6.61	6.68	6.76
furan	1 ^3^B_2_	π → π*	4.35	4.09	4.18	4.17
	1 ^3^A_1_	π → π*	5.65	5.48	5.54	5.48
pyrrole	1 ^3^B_2_	π → π*	4.73	4.43	4.52	4.48
	1 ^3^A_1_	π → π*	5.68	5.53	5.59	5.51
imidazole	1 ^3^A′	π → π*	4.77	4.67	4.69	4.69
	2 ^3^A′	π → π*	5.89	5.82	5.85	5.79
	1 ^3^A″	*n* → π*	6.46	6.33	6.36	6.37
	3 ^3^A′	π → π*	6.61	6.78	6.69	6.55
	4 ^3^A′	π → π*	7.06	7.33	7.24	7.42
	2 ^3^A″	*n* → π*	7.57	7.55	7.55	7.51
pyridine	1 ^3^A_1_	π → π*	4.47	4.18	4.28	4.25
	1 ^3^B_1_	*n* → π*	4.58	4.55	4.55	4.50
	1 ^3^B_2_	π → π*	4.94	4.93	4.95	4.86
	2 ^3^A_1_	π → π*	5.13	5.08	5.12	5.05
	1 ^3^A_2_	*n* → π*	5.46	5.60	5.52	5.46
	2 ^3^B_2_	π → π*	6.41	6.98	6.81	6.40
	3 ^3^B_2_	π → π*	7.23	7.15	7.18	7.83
	3 ^3^A_1_	π → π*	7.83	7.49	7.60	7.66
*s*-tetrazine	1 ^3^B_3u_	*n* → π*	1.64	1.97	1.94	1.89
	1 ^3^A_u_	*n* → π*	3.42	3.63	3.66	3.52
	1 ^3^B_1g_	*n* → π*	4.33	4.24	4.34	4.21
	1 ^3^B_1u_	π → π*	4.55	4.16	4.31	4.33
	1 ^3^B_2u_	π → π*	4.72	4.53	4.63	4.54
	1 ^3^B_2g_	*n* → π*	5.19	4.87	5.05	4.93
	2 ^3^A_u_	*n* → π*	5.03	5.24	5.24	5.03
	1 ^3^B_3g_	*n*,*n* → π*,π*	7.81	7.39	7.56	
	2 ^3^B_1u_	π → π*	5.51	5.43	5.48	5.38
	2 ^3^B_2g_	*n* → π*	6.11	6.09	6.18	6.04
	2 ^3^B_1g_	*n* → π*	6.55	6.47	6.57	6.60
	2 ^3^B_3u_	*n* → π*	6.72	6.75	6.81	6.53
	2 ^3^B_2u_	π → π*	6.39	7.28	7.05	7.36
formaldehyde	1 ^3^A_2_	*n* → π*	3.75	3.62	3.64	3.55
	1 ^3^A_1_	π → π*	6.04	5.64	5.79	5.83
acetone	1 ^3^A_2_	*n* → π*	4.10	4.18	4.12	4.05
	1 ^3^A_1_	π → π*	6.06	6.04	6.05	6.03
*p*-benzoquinone	1 ^3^B_1g_	*n* → π*	2.82	2.89	2.86	2.51
	1 ^3^A_u_	*n* → π*	2.82	2.91	2.89	2.62
	1 ^3^B_1u_	π → π*	2.93	3.01	3.07	2.96
	1 ^3^B_3g_	π → π*	3.40	3.54	3.54	3.41
formamide	1 ^3^A″	*n* → π*	5.64	5.27	5.40	5.36
	1 ^3^A′	π → π*	5.81	5.79	5.78	5.74
acetamide	1 ^3^A″	*n* → π*	5.50	5.38	5.41	5.42
	1 ^3^A′	π → π*	5.67	5.89	5.84	5.88
propanamide	1 ^3^A″	*n* → π*	5.55	5.42	5.45	5.45
	1 ^3^A′	π → π*	5.82	5.86	5.88	5.90

To expand our comparative analysis, we have incorporated CC3 results for four nucleobases (cytosine, thymine, uracil, and adenine) from the work of Kánnár and Szalay.^54^ While this reference did not include all excitations required for a comprehensive comparison, we have included available matching states in our analysis.

For completeness, we note that the NEVPT2 values reported within this work might differ slightly from those reported in the work reported by Schapiro et al.^23^ This slight difference is due to the handling of the symmetry in the ORCA program. The work done by Schapiro et al.^23^ utilized the ORCA 2.9 program released in 2012, and we are currently using ORCA 6.1 program released in 2025. The latest ORCA version enforces strict point group symmetry in optimizations, while the older version did not. This results in very slight differences in the final optimized geometries that are within the noise of the geometry optimization procedure. This very slight difference in geometry handling does change results for a handful of systems but with changes of less than 0.04 eV.

### 3.2 Singlet Excitations

Table 3 presents a comparison of the NEVPT2, NEVPT3, and NEVPT4 methods against the CC3 reference values. As noted previously, nine singlet excitations (from *s*-tetrazine, adenine, cytosine, thymine, and uracil) lack corresponding CC3 data and have consequently been excluded from the statistical analysis. This exclusion reduces the total count from 153 to 144 excitations, as reflected in Table 3.

**Table 3:** Statistical Analysis of Singlet Excitation Energies with Respect to CC3 Reported in eV^a^

	NEVPT2	NEVPT3	NEVPT4
count	144	144	144
ME	–0.11 (0.25)	0.15 (0.25)	0.02 (0.14)
SD	0.32 (0.22)	0.27 (0.18)	0.20 (0.14)

a Results are presented as “signed (unsigned),” where count is the total number of values used, ME is mean error, and SD is standard deviation.

The results in Table 3 demonstrate the expected progression in accuracy across the NEVPT series. Notably, the signed mean errors reveal a clear trend: −0.11 eV (NEVPT2), 0.15 eV (NEVPT3), and 0.02 eV (NEVPT4). This observed accuracy hierarchy NEVPT3 < NEVPT2 < NEVPT4 aligns with theoretical expectations and highlights the fact that stopping at third-order perturbation theory is usually not an optimal choice, especially given that the extra effort to proceed to fourth order (at least with an excitation space restricted to the FOIS) is not high.

To facilitate a direct comparison with previously reported CASPT2 values,^23^ we present in Table 4 a statistical analysis of singlet excitation energies relative to CC3, excluding the nucleobase data to maintain consistency with the earlier study. The table reports signed and unsigned errors (mean error, ME, and standard deviation, SD, in eV) for NEVPT2, NEVPT3, NEVPT4, and CASPT2.

**Table 4:** Statistical Analysis of Singlet Excitation Energies with Respect to CC3 Reported in eV Includes Previously Reported CASPT2 Results^a^

	NEVPT2	NEVPT3	NEVPT4	CASPT2
count	121	121	121	121
ME	–0.12 (0.28)	0.14 (0.26)	0.00 (0.14)	–0.17 (0.21)
SD	0.33 (0.22)	0.28 (0.18)	0.20 (0.15)	0.20 (0.16)

a Results are presented as “signed (unsigned),” where count is the total number of values used, ME is mean error, and SD is standard deviation.

The data reveal that NEVPT4 outperforms CASPT2 in terms of mean error, despite CASPT2 exhibiting a marginally smaller standard deviation. This difference in SD is not statistically significant and does not detract from the overall superior performance of NEVPT4. Furthermore, the minimal variation in ME and SD between Tables 3 and 4 suggests that the inclusion or exclusion of nucleobases has a negligible impact on the comparative assessment of these methods.

For further analysis of the NEVPT methodologies for the singlet excitation energies, we have prepared histogram plots, as shown in Figure 1. Figure 1 provides a detailed visualization of the singlet VEE errors relative to the CC3. The NEVPT2 results exhibit a broad distribution that deviates from an ideal Gaussian profile, particularly in the negative Δ*E* region. This distribution sharpens progressively with higher-order perturbative corrections: NEVPT3 shows to be reduced. It is pleasing to observe that NEVPT4 shows a near-Gaussian distribution. Notably, the systematic shifts in mean error are clearly reflected in these distributions: NEVPT2 is slightly biased toward negative errors, NEVPT3 is significantly biased toward positive errors, while NEVPT4 exhibits almost no bias.

**Figure 1:**
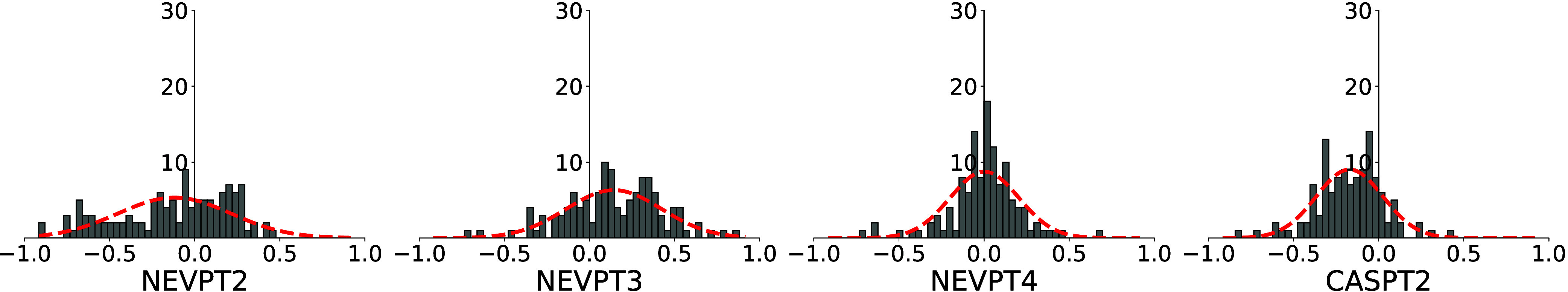
Histogram of singlet vertical excitation energies with the CC3 reference. The CASPT2 data shown is the previously reported result by Schapiro et al.^23^

Figure 2 presents correlation plots comparing each NEVPT method against the CC3 reference, including the corresponding coefficients of determination (*R* ^2^). Consistent with the error distribution analysis (Figure 1), NEVPT4 demonstrates superior agreement with CC3, as evidenced by its higher *R* ^2^ value, 0.985. This improvement in correlation with increasing perturbation order (NEVPT2 → NEVPT4) reflects the enhanced theoretical accuracy of the higher-order methods.

**Figure 2:**
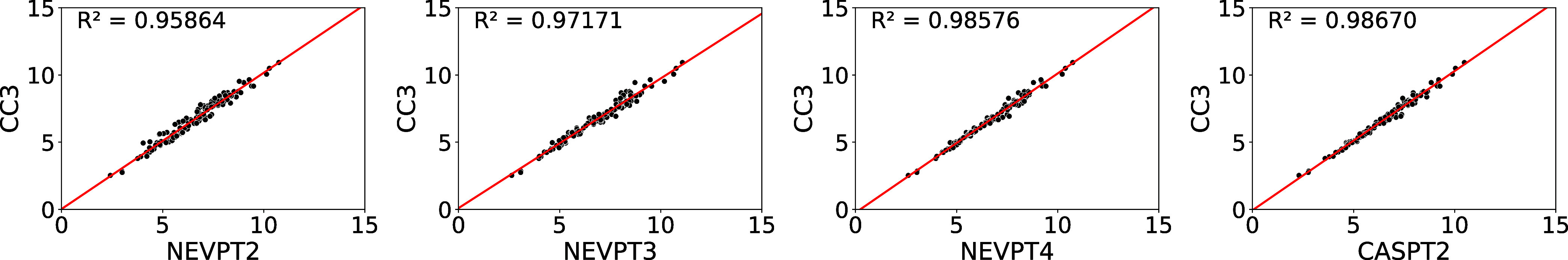
Correlation plots of singlet vertical excitation energies with the CC3 reference including regression lines and coefficient of determination (*R* ^2^). The CASPT2 data shown is the previously reported result by Schapiro et al.^23^

Overall, the NEVPT4 results exhibit a more balanced distribution about the regression line compared with NEVPT2 and NEVPT3, which show slight deviations. This symmetric distribution further confirms NEVPT4’s improved treatment of diverse electronic excitations within the singlet excitations for this benchmark set.

#### 3.2.1 π → π* Excitations

To gain deeper insight into the performance of the NEVPT methods, we analyze the singlet excitation energies based on their electronic character, starting with π → π* transitions. Our data set contains 85 π → π* VEEs, excluding nucleobases for consistency. Figure 3 compares the error distributions for π → π* transitions (yellow) against the complete benchmark set (gray), while Table 5 provides the corresponding statistical analysis.

**Figure 3:**
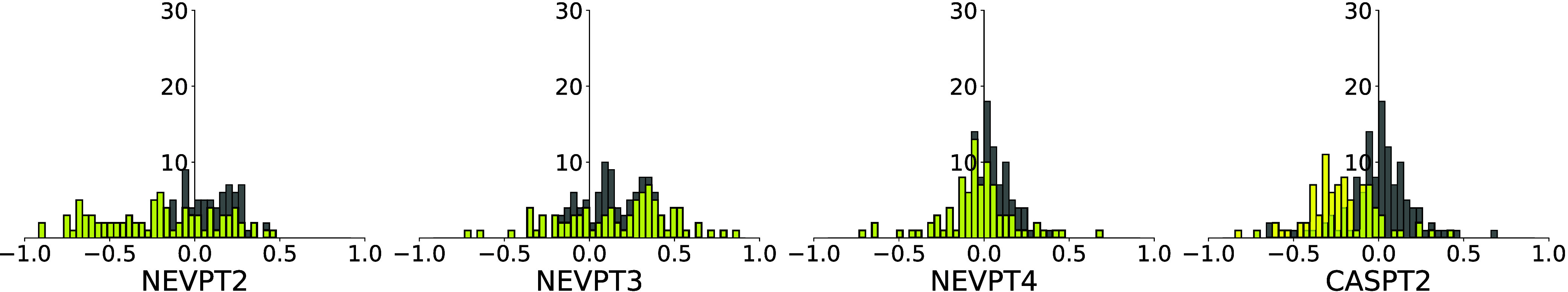
Histogram of the π → π* singlet vertical excitation energies with the CC3 reference in yellow. Statistics of all energies are shown in gray. The CASPT2 data shown is the previously reported result by Schapiro et al.^23^

**Table 5:** Statistical Analysis of π → π* Singlet Excitation Energies with Respect to CC3 Reported in eV, Including Previously Reported CASPT2 Results^a^

	NEVPT2	NEVPT3	NEVPT4	CASPT2
count	85	85	85	85
ME	–0.22 (0.33)	0.16 (0.30)	–0.04 (0.15)	–0.22 (0.25)
SD	0.34 (0.23)	0.32 (0.19)	0.22 (0.16)	0.21 (0.17)

a Results are presented as “signed (unsigned),” where count is the total number of values used, ME is mean error, and SD is standard deviation.

Both the visualization and quantitative metrics demonstrate NEVPT4’s superior performance for π → π* excitations, as shown in two results: first, a more concentrated error distribution (see Figure 3, yellow vs gray bars) and, second, the lowest mean error compared to the other methods that were tested (see Table 5). This improved accuracy reflects NEVPT4’s enhanced treatment of electron correlation in conjugated systems, particularly for delocalized excitations where dynamic correlation effects are significant. The signed quantitative data also reveals a clear, systematic progression across perturbation orders: NEVPT2 demonstrates a negative mean error of −0.22 eV, NEVPT3 exhibits overcorrection with a positive bias of +0.16 eV, and NEVPT4 attains near-balanced performance with a slight negative deviation of −0.04 eV, approaching zero. It has been observed that NEVPT methods outperform the previously reported CASPT2 method, which has an error of −0.22 eV. It is important to acknowledge that the NEVPT2, NEVPT4, and CASPT2 methodologies appear to underestimate the energies.

#### 3.2.2 *n* → π* Excitations

Turning to *n* → π* transitions, our data set comprises 33 VEEs, excluding nucleobases to maintain consistency with previous analyses. As shown in Figure 4 and Table 6, the NEVPT methods overestimate these excitations, with all three perturbation orders exhibiting positive mean deviations relative to the CC3 reference. This stands in contrast to CASPT2, which maintains its characteristic underestimation trend for these transitions (Table 6).

**Table 6:** Statistical Analysis of *n* → π* Singlet Excitation Energies with Respect to CC3 Reported in eV Includes Previously Reported CASPT2 Results^a^

	NEVPT2	NEVPT3	NEVPT4	CASPT2
count	33	33	33	33
ME	0.10 (0.14)	0.09 (0.14)	0.11 (0.11)	–0.07 (0.10)
SD	0.14 (0.10)	0.14 (0.08)	0.10 (0.09)	0.11 (0.08)

a Results are presented as “signed (unsigned),” where count is the total number of values used, ME is mean error, and SD is standard deviation.

**Figure 4:**
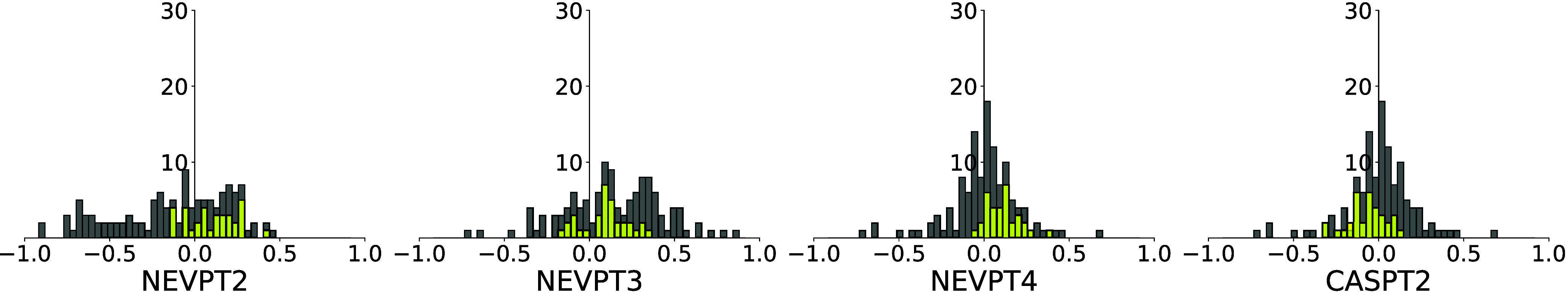
Histogram of the *n* → π* singlet vertical excitation energies with the CC3 reference in yellow. Statistics of all energies are shown in gray. The CASPT2 data shown is the previously reported result by Schapiro et al.^23^

The histogram distributions (Figure 4) reveal a consistent positive shift for all NEVPT methods, suggesting that the perturbative treatment of lone-pair to π* excitations introduces a distinct error not observed in π → π* transitions. Notably, while the magnitude of deviation decreases with higher-order perturbation, the fundamental bias persists across the NEVPT series. This behavior highlights the differential treatment of correlation effects in *n* → π* versus π → π* excitations within perturbation theory.

### 3.3 Triplet Excitations

We now turn to analysis of triplet excitations. As established in previous studies by Schreiber et al.^1^ and Schapiro et al.,^23^ triplet excitation classes generally exhibit better agreement with CC3 reference values due to their dominant single-reference character within multireference calculations.

The results for triplet excitations are summarized in Figures 5 and 6, which present histograms and correlation plots, respectively. The histograms (Figure 5) reveal a narrowing of the distribution with increasing perturbation order, mirroring the trend observed for singlet excitations. Similarly, the correlation plots (Figure 6) show comparable behavior, though the coefficient of determination (*R* ^2^) for triplet excitations is notably closer to unity, with NEVPT4 achieving an *R* ^2^ of 0.990.

**Figure 5:**
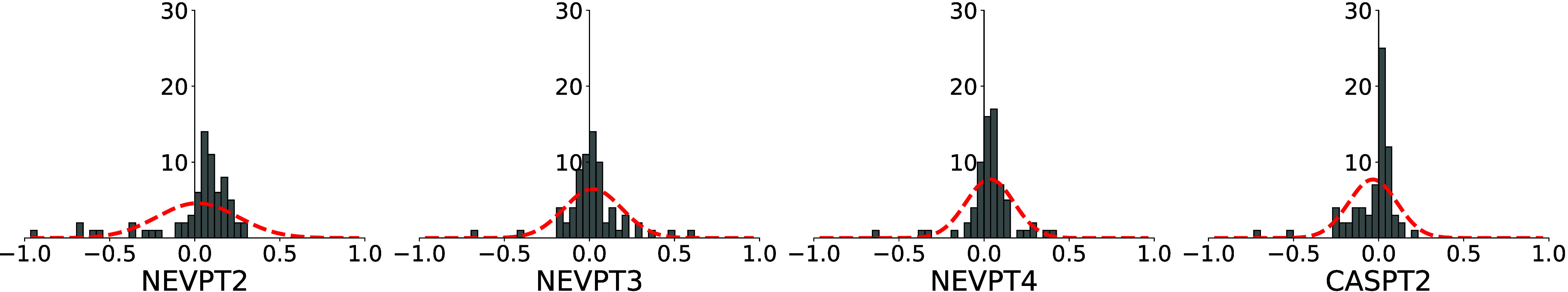
Histogram of triplet vertical excitation energies with the CC3 reference. The CASPT2 data shown is the previously reported result by Schapiro et al.^23^

**Figure 6:**
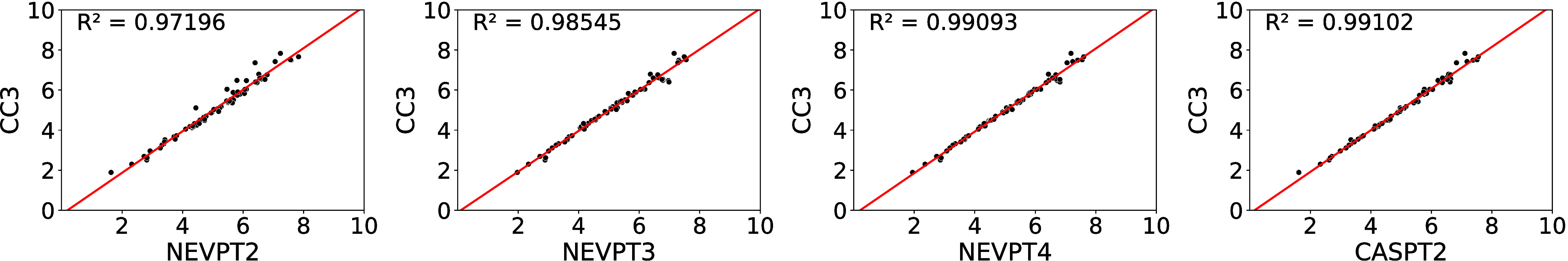
Correlation plots of triplet vertical excitation energies with the CC3 reference including regression lines and coefficient of determination (*R* ^2^). The CASPT2 data shown is the previously reported result by Schapiro et al.^23^

The mean errors and standard deviations, compiled in Table 7, further illustrate this trend. NEVPT methods consistently overestimate excitation energies with mean errors of 0.02, 0.02, and 0.03 eV for NEVPT2, NEVPT3, and NEVPT4, respectively. In contrast, CASPT2 slightly underestimates them (mean error: −0.03 eV). However, due to the strong single-reference character of triplet excitations, the absolute deviations for all methods remain very small, resulting in minimal differences between the approaches.

**Table 7:** Statistical Analysis of Triplet Excitation Energies with Respect to CC3 Reported in eV Includes Previously Reported CASPT2 Results^a^

	NEVPT2	NEVPT3	NEVPT4	CASPT2
count	71	71	71	71
ME	0.02 (0.16)	0.02 (0.11)	0.03 (0.09)	–0.03 (0.09)
SD	0.24 (0.17)	0.17 (0.14)	0.14 (0.11)	0.14 (0.12)

a Results are presented as “signed (unsigned),” where count is the total number of values used, ME is mean error, and SD is standard deviation.

As previously reported by Schapiro et al.,^23^ the most significant deviation in NEVPT2 is observed for the 2 ^3^B_2u_ excitation of *s*-tetrazine, attributable to an inadequate description of the zeroth-order wave function. Additional studies have been conducted by Angeli et al.,^49^ which demonstrate that the inclusion of additional π* virtual orbitals within the active space enhances the description of π → π* excitations for the *s*-tetrazine molecule. However, as the perturbation order increases for this excitation, the deviation becomes less pronounced, demonstrating a proper correction with perturbation expansion. Nevertheless, in contrast to the 2 ^3^B_2u_ excitation of *s*-tetrazine, the 3 ^3^B_2_ excitation of pyridine demonstrates consistent deviation across perturbation expansion, exhibiting differences of −0.60, −0.68, and −0.65 eV for NEVPT2, NEVPT3, and NEVPT4, respectively. This observation indicates that, under certain conditions, the perturbative expansion cannot serve to rectify the initial error in the zeroth-order wave function.

## 4 Conclusions

In this work, we demonstrate the accuracy of the FIC-NEVPT4­(SD) methodology in computing vertical electronic excitation energies by using the Thiel benchmarking set. A total of 225 vertical excitation energies were calculated, covering excitation classes such as π → π*, *n* → π*, and σ → π*. To ensure a direct and comprehensive comparison with the CC3 reference values reported by Schreiber et al.,^1^ we employed consistent active space selections, state averaging, and basis sets across all calculations. Previous investigations by our group on NEVPT2 also explored the influence of canonicalization procedures on this test set; however, for this specific case, the differences between canonicalization schemes were negligible. Thus, only the state-specific canonicalization is reported in the main work, while the state average is reported in the Supporting Information.

For singlet excitations, the FIC-NEVPT4­(SD) method outperforms both lower-order NEVPT approaches and the previously reported CASPT2, exhibiting a mean signed error of 0.14 compared to 0.21 (CASPT2), 0.28 (FIC-NEVPT2), and 0.26 (FIC-NEVPT3). The superior performance of FIC-NEVPT4­(SD) is further illustrated through histograms and correlation plots, which reveal a narrower error distribution and the highest *R* ^2^ value (0.985) among the tested methods. A detailed breakdown of singlet excitation types shows that *n* → π* transitions exhibit a consistent positive shift relative to π → π* excitations. While this shift persists across all NEVPT orders, its magnitude decreases with increasing perturbative treatment, suggesting that lone-pair to π* excitations introduce a distinct systematic error not present in π → π* transitions.

For triplet excitations, the performance differences between methods are less pronounced than those for singlets. Although FIC-NEVPT4­(SD) achieves an *R* ^2^ value close to unity in correlation plots, the mean error and standard deviation do not show significant improvement over lower-order NEVPT methods. This observation aligns with the inherently stronger single-reference character of triplet excitations, which reduces the impact of higher-order dynamic correlation effects.

In summary, this study establishes the accuracy and robustness of the FIC-NEVPT4­(SD) methodology for vertical excitation energies, demonstrating its advantages over lower-order perturbation theories while also identifying systematic trends in different excitation classes. In fact, the high accuracy reached by NEVPT4­(SD) might be considered slightly surprising given the approximations made, in particular, the neglect of triple excitations. While one might argue that the quadruple excitations and the renormalization term may cancel well to a large degree, the triple excitations should have a significant impact on the NEVPT4­(SD) results. One might argue that inside the active space, some of the higher-level excitations that are present in CC3 are already included. Whether all remaining triple excitations that are present in CC3 but not in NEVPT4­(SD) are actually negligible for the Thiel benchmark set or beyond remains to be seen. It seems clear from our results, however, that NEVPT4­(SD) is a cost-effective and accurate way to move beyond the limitations of second-order multireference perturbation theory such as NEVPT2 or CASPT2.

## Supplementary Material



## Notes

Detailed description of ORCA calculations of VEEs with and without symmetry. Full tables examining excitations utilizing the state-averaged canonicalization procedure and a more detailed description of the differing canonicalization procedures. Full documentation of all excitations calculated for every molecule examined. Geometrical coordinates in xyz format for all molecules in the Thiel set (PDF)

## References

[1] Schreiber M., Silva-Junior M. R., Sauer S. P. A., Thiel W. (2008). Benchmarks for Electronically
Excited States: CASPT2, CC2, CCSD, and CC3. J. Chem. Phys..

[2] Andersson K., Malmqvist P. A., Roos B. O., Sadlej A. J., Wolinski K. (1990). Second-Order
Perturbation Theory with a CASSCF Reference Function. J. Phys. Chem..

[3] Andersson K., Malmqvist P., Roos B. O. (1992). Second-order Perturbation Theory
with a Complete Active Space Self-consistent Field Reference Function. J. Chem. Phys..

[4] Koch H., Christiansen O., Jo/rgensen P., Sanchez de Merás A. M., Helgaker T. (1997). The CC3Model:
An Iterative Coupled Cluster Approach
Including Connected Triples. J. Chem. Phys..

[5] Hald K., Hättig C., Yeager D. L., Jørgensen P. (2000). Linear Response
CC2 Triplet Excitation Energies. Chem. Phys.
Lett..

[6] Christiansen O., Koch H., Jørgensen P. (1995). The Second-Order
Approximate Coupled
Cluster Singles and Doubles Model CC2. Chem.
Phys. Lett..

[7] Christiansen O., Koch H., Jørgensen P. (1995). Response Functions
in the CC3 Iterative
Triple Excitation Model. J. Chem. Phys..

[8] Hald K., Hättig C., Jørgensen P. (2000). Triplet Excitation Energies in the
Coupled Cluster Singles and Doubles Model Using an Explicit Triplet
Spin Coupled Excitation Space. J. Chem. Phys..

[9] Koch H., Jørgensen P. (1990). Coupled Cluster
Response Functions. J. Chem. Phys..

[10] Larsen H., Olsen J., Jørgensen P., Christiansen O. (2000). Full Configuration
Interaction Benchmarking of Coupled-Cluster Models for the Lowest
Singlet Energy Surfaces of N 2. J. Chem. Phys..

[11] Larsen H., Hald K., Olsen J., Jørgensen P. (2001). Triplet Excitation
Energies in Full Configuration Interaction and Coupled-Cluster Theory. J. Chem. Phys..

[12] Pecul M., Jaszuński M., Larsen H., Jørgensen P. (2000). Singlet Excited
States of Be 2. J. Chem. Phys..

[13] Christiansen O., Koch H., Jørgensen P., Olsen J. (1996). Excitation Energies
of H2O, N2 and C2 in Full Configuration Interaction and Coupled Cluster
Theory. Chem. Phys. Lett..

[14] Koch H., Christiansen O., Jørgensen P., Olsen J. (1995). Excitation Energies
of BH, CH2 and Ne in Full Configuration Interaction and the Hierarchy
CCS, CC2, CCSD and CC3 of Coupled Cluster Models. Chem. Phys. Lett..

[15] Cronstrand P., Christiansen O., Norman P., Ågren H. (2000). Theoretical
Calculations of Excited State Absorption. Phys.
Chem. Chem. Phys..

[16] Hald K., Jørgensen P., Breckenridge W. H., Jaszuński M. (2002). Calculation
of Ground and Excited State Potential Energy Curves of the MgAr Complex
Using the Coupled Cluster Approximate Triples Model CC3. Chem. Phys. Lett..

[17] Silva-Junior M. R., Schreiber M., Sauer S. P. A., Thiel W. (2008). Benchmarks
for Electronically
Excited States: Time-Dependent Density Functional Theory and Density
Functional Theory Based Multireference Configuration Interaction. J. Chem. Phys..

[18] Maier T. M., Bahmann H., Arbuznikov A. V., Kaupp M. (2016). Validation of Local
Hybrid Functionals for TDDFT Calculations of Electronic Excitation
Energies. J. Chem. Phys..

[19] Kempfer-Robertson E. M., Pike T. D., Thompson L. M. (2020). Difference Projection-after-Variation
Double-Hybrid Density Functional Theory Applied to the Calculation
of Vertical Excitation Energies. J. Chem. Phys..

[20] Liu X., Subotnik J. E. (2014). The Variationally
Orbital-Adapted Configuration Interaction
Singles (VOA-CIS) Approach to Electronically Excited States. J. Chem. Theory Comput..

[21] Helmich B., Hättig C. (2014). A Pair Natural
Orbital Based Implementation of ADC(2)-x:
Perspectives and Challenges for Response Methods for Singly and Doubly
Excited States in Large Molecules. Excit. States
Isol. Mol. Complex Environ..

[22] Harbach P. H. P., Wormit M., Dreuw A. (2014). The Third-Order
Algebraic Diagrammatic
Construction Method (ADC(3)) for the Polarization Propagator for Closed-Shell
Molecules: Efficient Implementation and Benchmarkinga). J. Chem. Phys..

[23] Schapiro I., Sivalingam K., Neese F. (2013). Assessment of N-Electron Valence
State Perturbation Theory for Vertical Excitation Energies. J. Chem. Theory Comput..

[24] Angeli C., Cimiraglia R., Evangelisti S., Leininger T., Malrieu J.-P. (2001). Introduction of
N-Electron Valence States for Multireference
Perturbation Theory. J. Chem. Phys..

[25] Angeli C., Cimiraglia R., Malrieu J.-P. (2001). N-Electron Valence State Perturbation
Theory: A Fast Implementation of the Strongly Contracted Variant. Chem. Phys. Lett..

[26] Angeli C., Cimiraglia R., Malrieu J.-P. (2002). N-Electron Valence State Perturbation
Theory: A Spinless Formulation and an Efficient Implementation of
the Strongly Contracted and of the Partially Contracted Variants. J. Chem. Phys..

[27] Franz M., Neese F., Richert S. (2022). Calculation
of Exchange Couplings
in the Electronically Excited State of Molecular Three-Spin Systems. Chem. Sci..

[28] Sarkar R., Loos P.-F., Boggio-Pasqua M., Jacquemin D. (2022). Assessing
the Performances of CASPT2 and NEVPT2 for Vertical Excitation Energies. J. Chem. Theory Comput..

[29] Gupta S. K., Rao S. V., Demeshko S., Dechert S., Bill E., Atanasov M., Neese F., Meyer F. (2023). Air-Stable Four-Coordinate
Cobalt­(Ii) Single-Ion Magnets: Experimental and Ab Initio Ligand Field
Analyses of Correlations between Dihedral Angles and Magnetic Anisotropy. Chem. Sci..

[30] Singh S. K., Eng J., Atanasov M., Neese F. (2017). Covalency and Chemical Bonding in
Transition Metal Complexes: An Ab Initio Based Ligand Field Perspective. Chem. Bond. State Art.

[31] Angeli C., Pastore M., Cimiraglia R. (2007). New Perspectives
in Multireference
Perturbation Theory: The n-Electron Valence State Approach. Theor. Chem. Acc..

[32] Angeli C., Cimiraglia R. (2014). Some Useful
Odds and Ends From the N-Electron Valence
State Perturbation Theory. J. Phys. Chem. A.

[33] Guo Y., Sivalingam K., Valeev E. F., Neese F. (2016). SparseMaps—A
Systematic Infrastructure for Reduced-Scaling Electronic Structure
Methods. III. Linear-Scaling Multireference Domain-Based Pair Natural
Orbital N-Electron Valence Perturbation Theory. J. Chem. Phys..

[34] Guo Y., Pavošević F., Sivalingam K., Becker U., Valeev E. F., Neese F. (2023). SparseMaps—A
Systematic Infrastructure for Reduced-Scaling Electronic Structure
Methods. VI. Linear-Scaling Explicitly Correlated N-Electron Valence
State Perturbation Theory with Pair Natural Orbital. J. Chem. Phys..

[35] Guo Y., Sivalingam K., Chilkuri V. G., Neese F. (2025). Approximations of Density
Matrices in N-Electron Valence State Second-Order Perturbation Theory
(NEVPT2). III. Large Active Space Calculations with Selected Configuration
Interaction Reference. J. Chem. Phys..

[36] Park J. W. (2019). Analytical
Gradient Theory for Strongly Contracted (SC) and Partially Contracted
(PC) N-Electron Valence State Perturbation Theory (NEVPT2). J. Chem. Theory Comput..

[37] Sokolov A. Yu., Guo S., Ronca E., Chan G. K.-L. (2017). Time-Dependent N-Electron Valence
Perturbation Theory with Matrix Product State Reference Wavefunctions
for Large Active Spaces and Basis Sets: Applications to the Chromium
Dimer and All-Trans Polyenes. J. Chem. Phys..

[38] Guo Y., Pernal K. (2025). Approximation to Second
Order N-Electron Valence State
Perturbation Theory: Limiting the Wave Function within Singles. J. Chem. Theory Comput..

[39] Bertels L. W., Lee J., Head-Gordon M. (2019). Third-Order
Mo̷ller-Plesset Perturbation Theory
Made Useful? Choice of Orbitals and Scaling Greatly Improves Accuracy
for Thermochemistry, Kinetics, and Intermolecular Interactions. J. Phys. Chem. Lett..

[40] Grimme S., Parac M., Waletzke M. (2001). On the Importance
of Third- and Fourth-Order
Corrections in Multi-Reference Mo̷ller-Plesset Theory. Chem. Phys. Lett..

[41] Kempfer E. M., Sivalingam K., Neese F. (2025). Efficient Implementation of Approximate
Fourth Order N-Electron Valence State Perturbation Theory. J. Chem. Theory Comput..

[42] Sivalingam K., Krupicka M., Auer A. A., Neese F. (2016). Comparison of Fully
Internally and Strongly Contracted Multireference Configuration Interaction
Procedures. J. Chem. Phys..

[43] Angeli C. (2009). On the Nature
of the π → Π* Ionic Excited States: The V State
of Ethene as a Prototype. J. Comput. Chem..

[44] Angeli C. (2010). An Analysis
of the Dynamic σ Polarization in the V State of Ethene. Int. J. Quantum Chem..

[45] Angeli C., Pastore M. (2011). The Lowest Singlet
States of Octatetraene Revisited. J. Chem. Phys..

[46] Pastore M., Angeli C., Cimiraglia R. (2006). An Application of Second and Third-Order
n-Electron Valence State Perturbation Theory to the Calculation of
the Vertical Electronic Spectrum of Furan. Chem.
Phys. Lett..

[47] Havenith R. W., Taylor P. R., Angeli C., Cimiraglia R., Ruud K. (2004). Calibration of the N-Electron Valence State Perturbation Theory Approach. J. Chem. Phys..

[48] Pastore M., Angeli C., Cimiraglia R. (2006). The Vertical
Electronic Spectrum
of Pyrrole: A Second and Third Order n-Electron Valence State Perturbation
Theory Study. Chem. Phys. Lett..

[49] Angeli C., Cimiraglia R., Cestari M. (2009). A Multireference N-Electron Valence
State Perturbation Theory Study of the Electronic Spectrum of s-Tetrazine. Theor. Chem. Acc..

[50] Angeli C., Borini S., Ferrighi L., Cimiraglia R. (2005). Ab Initio
N-Electron Valence State Perturbation Theory Study of the Adiabatic
Transitions in Carbonyl Molecules: Formaldehyde, Acetaldehyde, and
Acetone. J. Chem. Phys..

[51] Angeli C., Borini S., Cimiraglia R. (2004). An Application of Second-Order n-Electron
Valence State Perturbation Theory to the Calculation of Excited States. Theor. Chem. Acc..

[52] Neese F., Wennmohs F., Becker U., Riplinger C. (2020). The ORCA Quantum
Chemistry Program Package. J. Chem. Phys..

[53] Lechner M. H., Papadopoulos A., Sivalingam K., Auer A. A., Koslowski A., Becker U., Wennmohs F., Neese F. (2024). Code Generation in
ORCA: Progress, Efficiency and Tight Integration. Phys. Chem. Chem. Phys..

[54] Kánnár D., Szalay P. G. (2014). Benchmarking
Coupled Cluster Methods on Singlet Excited
States of Nucleobases. J. Mol. Model..

