# Supplementary material for: Evaluation of Approximate Fourth-Order N‑Electron Valence Perturbation Theory (NEVPT4(SD)) for the Excited States of Organic Molecules
Source: J Chem Theory Comput. 2025 Nov 20;21(23):12185–96. doi: 10.1021/acs.jctc.5c01685 (PMC12874378; doi:10.1021/acs.jctc.5c01685)
Supplement: Supplementary file 1 [file ct5c01685_si_001.pdf]

Supplementary Information: Evaluation of Approximate Fourth Order N-Electron Valence Perturbation Theory (NEVPT4(SD)) for the Excited States of Organic Molecules

Emily M. Kempfer,[1] Kantharuban Sivalingam[1] and Frank Neese[1]*

1- Max Planck Institut für Kohlenforschung

Kaiser-Wilhelm Platz 1

D-45472 Mülheim an der Ruhr, Germany

Email: Neese@kofo.mpg.de

Table of Contents

S1 Computation of VEEs **2**

S1.1 Same Symmetry **3**

S1.2 Different Symmetry **3**

S2 State Averaged Canonicalization Benchmarking Set **4**

S3 Documentation of Benchmarking Results **16**

S3.1 Unsaturated Aliphatic Hydrocarbons **16**

S3.1.1 Ethene 16
S3.1.2 E-Butadiene 16
S3.1.3 E-Hexatriene 17
S3.1.4 E-Octatetraene 17
S3.1.5 Cyclopropene 18
S3.1.6 Cyclopentadiene 18
S3.1.7 Norbornadiene 19

S3.2 Aromatic Hydrocarbons and Heterocycles **19**

S3.2.1 Benzene 19
S3.2.2 Naphthalene 20
S3.2.3 Furan 21
S3.2.4 Pyrrole 21
S3.2.5 Imidazole 22
S3.2.6 Pyridine 23
S3.2.7 Pyrazine 24
S3.2.8 Pyrimidine 24
S3.2.9 Pyridazine 25
S3.2.10 s-Triazine 25
S3.2.11 s-Tetrazine 26

S3.3 Aldehydes, Ketones and Amides **27**

S3.3.1 Formaldehyde 27
S3.3.2 Acetone 27
S3.3.3 p-Benzoquinone 28
S3.3.4 Formamide 28
S3.3.5 Acetamide 29
S3.3.6 Propanamide 29

S3.4 Nucelobases **30**

S3.4.1 Cytosine 30
S3.4.2 Thymine 30
S3.4.3 Uracil 31
S3.4.3 Adenine 31

S4 Documentation of Geometric Coordinates **32**

S4.1 Unsaturated Aliphatic Hydrocarbons **32**
S4.1.1 Ethene 32
S4.1.2 E-Butadiene 32
S4.1.3 E-Hexatriene 32
S4.1.4 E-Octatetraene 33
S4.1.5 Cyclopropene 33
S4.1.6 Cyclopentadiene 33
S4.1.7 Norbornadiene 34

S4.2 Aromatic Hydrocarbons and Heterocycles **34**
S4.2.1 Benzene 34
S4.2.2 Naphthalene 34
S4.2.3 Furan 35
S4.2.4 Pyrrole 35
S4.2.5 Imidazole 35
S4.2.6 Pyridine 35
S4.2.7 Pyrazine 37
S4.2.8 Pyrimidine 37
S4.2.9 Pyridazine 37
S4.2.10 s-Triazine 37
S4.2.11 s-Tetrazine 39

S4.3 Aldehydes, Ketones and Amides **39**
S4.3.1 Formaldehyde 39
S4.3.2 Acetone 39
S4.3.3 p-Benzoquinone 39
S4.3.4 Formamide 40
S4.3.5 Acetamide 40
S4.3.6 Propanamide 40

S4.4 Nucelobases **40**
S4.4.1 Cytosine 40
S4.4.2 Thymine 41
S4.4.3 Uracil 41
S4.4.3 Adenine 41

S1 Computation of VEEs

The computation of vertical excitation energies (VEEs) is briefly outlined in the Computational Details section of the main text. Here, we provide a more detailed description for researchers who may wish to reproduce or extend this dataset.

In general, there are two possible scenarios when computing VEEs:

(1) Ground state and excited state have <u>same symmetry</u>

(2) Excited state is <u>different in symmetry</u> than the ground state

These two approaches can be illustrated using the two lowest singlet excited states of E-butadiene as examples.

S1.1 Same Symmetry

First, consider the case where the ground and excited states belong to the same irreducible representation. In this situation, the ground-state energy from a state-averaged (SA) calculation is used as the zeroth-order reference.

For E-butadiene, the ground state has $1A_g$ symmetry. Thus, when computing the $2A_g$ excited state, we employ a single state-averaged calculation over the relevant A_g states.

Below is an example ORCA input for the CASSCF calculation:

```
!TZVP NoFrozenCore NoRI PAL4
!UseSym AutoAux
# PModel default guess happens to produce the correct active space here.
%casscf
  nel 4 norb 4
  mult 1
  irrep 0
  nroots 3
  maxiter 200
  shiftup 2.0
  shiftdn 2.0
  etol 1e-9
  gtol 1e-5
end

* xyzfile 0 1 E-butadiene.xyz
```

Listing S1 Example input for computing the state-averaged A_g states of E-butadiene, used for the VEE of the 2 A_g state.

From this calculation, we obtain the state-averaged energies of the relevant A_g states. The VEE for the 2 A_g state is then computed as:

$$\Delta E_{2Ag} = E_{2Ag}^{SA} - E_{1Ag}^{SA} \tag{1}$$

S1.2 Different Symmetry

Now consider the case where the excited state belongs to a different irreducible representation than the ground state. In this scenario, the required energies cannot be obtained from a single calculation. Instead, at least two separate calculations are required:

(1) A state-specific (SS) calculation for the ground state.

(2) A state-averaged (SA) calculation for the excited-state manifold

For E-butadiene, the $1B_u$ excited state differs in symmetry from the $1A_g$ ground state. Thus, we first compute the ground state using a state-specific calculation:

```
!TZVP NoFrozenCore NoRI PAL4
!UseSym AutoAux

%casscf
  nel 4 norb 4
  mult 1
  irrep 0
  nroots 1
  maxiter 200
  shiftup 2.0
  shiftdn 2.0
  etol 1e-9
  gtol 1e-5
end

* xyzfile 0 1 E-butadiene.xyz
```

Listing S2 Example ORCA input for the state-specific calculation of the $1A_g$ ground state.

Next, we perform a state-averaged calculation for the B_u symmetry:

```
!TZVP NoFrozenCore NoRI PAL4
!UseSym AutoAux
```

```
%casscf
  nel 4 norb 4
  mult 1
  irrep 3
  nroots 2
  maxiter 200
  shiftup 2.0
  shiftdn 2.0
  etol 1e-9
  gtol 1e-5
end

* xyzfile 0 1 E-butadiene.xyz
```

Listing S3 Example input for computing the state-averaged B_u states of E-butadiene.

From these two calculations, we obtain the state-specific ground-state energy ($1A_g$) and the state-averaged excited-state energy ($1B_u$). The VEE for the $1B_u$ state is then calculated as:

$$\Delta E_{1Bu} = E_{1Bu}^{SA} - E_{1Ag}^{SS} \quad (2)$$

It is important to note the distinction between Eqs. (1) and (2): in Eq. (1), the ground-state energy is state-averaged, whereas in Eq. (2) it is state-specific.

S2 State Averaged Canonicalization Benchmarking Set

As described in the Computational Details section of the main text, two canonicalization strategies were evaluated for this benchmarking set:

(1) The more accurate state-specific approach, in which orbital energies and orbitals are obtained by diagonalizing the state-specific Fock operator (called within orca as canonstep=1), and

(2) the approximate approach, where orbital energies are taken as the diagonal elements of the state-specific Fock operator while the orbitals remain state-averaged and pseudo-canonicalized.

The differences between these two approaches were found to be negligible, shown in table S1 for the singlet excitations. For this reason, only the fully state-specific canonicalization (option 1) is presented in detail in the main article. For completeness, the results obtained with the approximate approach (option 2) are provided below, though we emphasize that the discrepancies between the two methods are insignificant.

Table S1 Signed Mean Error (ME) and Standard Deviation (SD) of the two canonicalization steps for singlet excitations

	State Specific Canonicalization (reported in main article)			*State Averaged Canonicalization*		
	NEVPT2	*NEVPT3*	*NEVPT4*	*NEVPT2*	*NEVPT3*	*NEVPT4*
Count	*121*	*121*	*121*	*121*	*121*	*121*
ΔME	*-0.12*	*0.15*	*0.00*	*-0.12*	*0.14*	*0.00*
ΔSD	*0.34*	*0.28*	*0.20*	*0.33*	*0.28*	*0.20*

Table S2 Singlet Vertical Excitation Energies ΔE (eV) (canonicalization is state averaged)

Molecule	*State*	*Type*	*NEVPT2*	*NEVPT3*	*NEVPT4*	*CC3*
Ethene	$1\ ^1B_{1u}$	$\pi \rightarrow \pi^*$	8.64	8.74	8.45	8.37
E-Butadiene	$1\ ^1B_u$	$\pi \rightarrow \pi^*$	6.14	6.93	6.56	6.58
	$2\ ^1A_g$	$\pi \rightarrow \pi^*$	6.86	6.57	6.63	6.77
all-E-Hexatriene	$1\ ^1B_u$	$\pi \rightarrow \pi^*$	4.84	5.94	5.53	5.58
	$2\ ^1A_g$	$\pi \rightarrow \pi^*$	5.56	5.44	5.47	5.72
all-E-Octatetraene	$2\ ^1A_g$	$\pi \rightarrow \pi^*$	4.73	4.64	4.68	4.97
	$1\ ^1B_u$	$\pi \rightarrow \pi^*$	4.04	5.30	4.87	4.94
	$2\ ^1B_u$	$\pi \rightarrow \pi^*$	5.86	5.85	5.87	6.06
	$3\ ^1A_g$	$\pi \rightarrow \pi^*$	5.97	7.13	6.66	6.50
	$4\ ^1A_g$	$\pi \rightarrow \pi^*$	6.67	6.46	6.51	6.81
	$3\ ^1B_u$	$\pi \rightarrow \pi^*$	8.35	8.20	8.25	7.91
Cyclopropene	$1\ ^1B_1$	$\sigma \rightarrow \pi^*$	6.84	6.95	6.91	6.90
	$1\ ^1B_2$	$\pi \rightarrow \pi^*$	7.03	7.40	7.14	7.10
Cyclopentadiene	$1\ ^1B_2$	$\pi \rightarrow \pi^*$	5.16	6.05	5.66	5.73
	$2\ ^1A_1$	$\pi \rightarrow \pi^*$	6.78	6.53	6.57	6.61
	$3\ ^1A_1$	$\pi \rightarrow \pi^*$	8.27	9.06	8.55	8.69
Norbornadiene	$1\ ^1A_2$	$\pi \rightarrow \pi^*$	5.02	6.02	5.64	5.64
	$1\ ^1B_2$	$\pi \rightarrow \pi^*$	5.77	7.03	6.43	6.49
	$2\ ^1B_2$	$\pi \rightarrow \pi^*$	7.00	8.16	7.59	7.64
	$2\ ^1A_2$	$\pi \rightarrow \pi^*$	7.07	8.20	7.69	7.71
Benzene	$1\ ^1B_{2u}$	$\pi \rightarrow \pi^*$	5.23	5.00	5.06	5.07
	$1\ ^1B_{1u}$	$\pi \rightarrow \pi^*$	6.40	6.82	6.66	6.68

Molecule	State	Type	NEVPT2	NEVPT3	NEVPT4	CC3
	$1\,^1E_{1u}$	$\pi \rightarrow \pi^*$	7.11	7.75	7.39	7.45
	$2\,^1E_{2g}$	$\pi \rightarrow \pi^*$	8.42	8.17	8.24	8.43
Naphthalene	$1\,^1B_{3u}$	$\pi \rightarrow \pi^*$	4.38	4.25	4.30	4.27
	$1\,^1B_{2u}$	$\pi \rightarrow \pi^*$	4.36	5.28	4.99	5.03
	$2\,^1A_g$	$\pi \rightarrow \pi^*$	6.22	5.86	5.97	5.98
	$1\,^1B_{1g}$	$\pi \rightarrow \pi^*$	6.11	6.19	6.17	6.07
	$2\,^1B_{3u}$	$\pi \rightarrow \pi^*$	5.61	6.68	6.25	6.33
	$2\,^1B_{1g}$	$\pi \rightarrow \pi^*$	6.18	7.03	6.73	6.79
	$2\,^1B_{2u}$	$\pi \rightarrow \pi^*$	6.02	6.81	6.53	6.57
	$3\,^1A_g$	$\pi \rightarrow \pi^*$	6.84	6.70	6.77	6.90
	$3\,^1B_{2u}$	$\pi \rightarrow \pi^*$	7.82	8.70	8.31	8.44
	$3\,^1B_{3u}$	$\pi \rightarrow \pi^*$	7.92	7.78	7.84	8.12
Furan	$1\,^1B_2$	$\pi \rightarrow \pi^*$	6.38	7.03	6.71	6.60
	$2\,^1A_1$	$\pi \rightarrow \pi^*$	6.80	6.61	6.65	6.62
	$3\,^1A_1$	$\pi \rightarrow \pi^*$	8.44	8.98	8.60	8.53
Pyrrole	$2\,^1A_1$	$\pi \rightarrow \pi^*$	6.58	6.40	6.44	6.40
	$1\,^1B_2$	$\pi \rightarrow \pi^*$	6.74	7.10	6.92	6.71
	$3\,^1A_1$	$\pi \rightarrow \pi^*$	8.22	8.54	8.29	8.17
Imidazole	$1\,^1A''$	$n \rightarrow \pi^*$	6.94	6.81	6.83	6.82
	$2\,^1A'$	$\pi \rightarrow \pi^*$	6.83	6.72	6.73	6.58
	$3\,^1A'$	$\pi \rightarrow \pi^*$	6.90	7.21	7.04	7.10
	$2\,^1A''$	$n \rightarrow \pi^*$	8.02	7.98	7.97	7.93
	$4\,^1A'$	$\pi \rightarrow \pi^*$	8.45	8.78	8.49	8.45

Molecule	*State*	*Type*	*NEVPT2*	*NEVPT3*	*NEVPT4*	*CC3*
Pyridine	$1\ ^1B_2$	$\pi \rightarrow \pi^*$	5.33	5.10	5.18	5.15
	$1\ ^1B_1$	$n \rightarrow \pi^*$	5.26	5.14	5.14	5.05
	$1\ ^1A_2$	$n \rightarrow \pi^*$	5.46	5.62	5.53	5.50
	$2\ ^1A_1$	$\pi \rightarrow \pi^*$	7.08	7.34	7.28	6.85
	$3\ ^1A_1$	$\pi \rightarrow \pi^*$	7.23	8.06	7.62	7.70
	$2\ ^1B_2$	$\pi \rightarrow \pi^*$	7.11	8.09	7.60	7.59
	$4\ ^1A_1$	$\pi \rightarrow \pi^*$	8.07	8.05	8.06	8.68
	$3\ ^1B_2$	$\pi \rightarrow \pi^*$	8.53	8.30	8.35	8.77
Pyrazine	$1\ ^1B_{3u}$	$n \rightarrow \pi^*$	4.20	4.36	4.34	4.24
	$1\ ^1A_u$	$n \rightarrow \pi^*$	4.95	5.27	5.19	5.05
	$1\ ^1B_{2u}$	$\pi \rightarrow \pi^*$	5.30	5.03	5.08	5.02
	$1\ ^1B_{2g}$	$n \rightarrow \pi^*$	5.86	5.68	5.75	5.74
	$1\ ^1B_{1g}$	$n \rightarrow \pi^*$	6.79	6.86	6.87	6.75
	$1\ ^1B_{1u}$	$\pi \rightarrow \pi^*$	6.76	7.25	7.04	7.07
	$2\ ^1B_{1u}$	$\pi \rightarrow \pi^*$	7.72	8.53	7.98	8.06
	$2\ ^1B_{2u}$	$\pi \rightarrow \pi^*$	7.44	8.31	7.87	8.05
	$1\ ^1B_{3g}$	$\pi \rightarrow \pi^*$	8.73	8.46	8.55	8.77
	$2\ ^1A_g$	$\pi \rightarrow \pi^*$	8.86	8.51	8.61	8.69
Pyrimidine	$1\ ^1B_1$	$n \rightarrow \pi^*$	4.52	4.58	4.57	4.50
	$1\ ^1A_2$	$n \rightarrow \pi^*$	4.81	5.02	4.98	4.93
	$1\ ^1B_2$	$\pi \rightarrow \pi^*$	5.61	5.32	5.39	5.36
	$2\ ^1A_1$	$\pi \rightarrow \pi^*$	7.42	7.59	7.50	7.06
	$2\ ^1B_2$	$\pi \rightarrow \pi^*$	7.50	8.44	7.94	8.01

Molecule	State	Type	NEVPT2	NEVPT3	NEVPT4	CC3
	$3\ ^1A_1$	π → π*	7.74	8.46	8.06	7.74
Pyridazine	$1\ ^1B_1$	n → π*	3.91	4.08	4.03	3.92
	$1\ ^1A_2$	n → π*	4.58	4.67	4.65	4.49
	$2\ ^1A_1$	π → π*	5.47	5.12	5.24	5.22
	$2\ ^1A_2$	n → π*	5.89	5.87	5.86	5.74
	$2\ ^1B_1$	n → π*	6.69	6.68	6.66	6.41
	$1\ ^1B_2$	π → π*	7.35	7.78	7.61	6.93
	$2\ ^1B_2$	π → π*	7.25	8.08	7.62	7.55
	$3\ ^1A_1$	π → π*	7.37	8.33	7.76	7.82
s-Triazine	$1\ ^1A_1''$	n → π*	4.65	5.08	4.99	4.78
	$1\ ^1A_2''$	n → π*	4.88	4.85	4.90	4.76
	$1\ ^1E''$	n → π*	4.87	5.01	5.01	4.81
	$1\ ^1A_2'$	π → π*	5.92	5.63	5.75	5.71
	$2\ ^1A_1'$	π → π*	7.19	7.71	7.51	7.41
	$2\ ^1E''$	n → π*	7.97	7.89	7.93	7.80
	$1\ ^1E'$	π → π*	7.95	8.82	8.35	8.04
	$2\ ^1E'$	π → π*	9.03	8.73	8.80	9.44
s-Tetrazine	$1\ ^1B_{3u}$	n → π*	2.41	2.63	2.61	2.53
	$1\ ^1A_u$	π → π*	3.79	3.97	3.98	3.79
	$1\ ^1B_{1g}$	n → π*	5.16	5.04	5.12	4.97
	$1\ ^1B_{2u}$	π → π*	5.49	4.98	5.17	5.12
	$1\ ^1B_{2g}$	n → π*	5.53	5.20	5.34	5.34
	$1\ ^1B_{3g}$	n,n → π*,π*	6.30	6.37	6.40	

Molecule	State	Type	NEVPT2	NEVPT3	NEVPT4	CC3
	$2\,^1A_u$	π → π*	5.68	5.69	5.69	5.46
	$2\,^1B_{2g}$	n → π*	6.29	6.30	6.36	6.23
	$2\,^1B_{1g}$	n → π*	6.82	6.70	6.81	6.87
	$3\,^1B_{1g}$	n → π*	7.04	6.94	7.05	7.08
	$2\,^1B_{3u}$	n → π*	7.12	7.03	7.07	6.67
	$1\,^1B_{1u}$	π → π*	6.78	7.56	7.31	7.45
	$2\,^1B_{1u}$	π → π*	6.86	7.97	7.40	7.79
	$2\,^1B_{2u}$	π → π*	8.30	8.16	8.22	8.51
	$2\,^1B_{3g}$	π → π*	8.08	8.51	8.37	8.47
Formaldehyde	$1\,^1A_2$	n → π*	4.22	4.00	4.02	3.95
	$1\,^1B_1$	σ → π*	9.40	9.21	9.23	9.18
	$2\,^1A_1$	π → π*	8.66	10.32	9.13	9.53
Acetone	$1\,^1A_2$	n → π*	4.47	4.54	4.47	4.40
	$1\,^1B_1$	σ → π*	9.50	9.55	9.42	9.17
	$2\,^1A_1$	π → π*	9.22	9.50	9.16	9.65
p-Benzoquinone	$1\,^1A_u$	n → π*	2.99	3.08	3.05	2.85
	$1\,^1B_{1g}$	n → π*	3.00	3.07	3.03	2.75
	$1\,^1B_{3g}$	π → π*	4.28	4.98	4.82	4.59
	$1\,^1B_{1u}$	π → π*	4.82	6.02	5.61	5.62
	$1\,^1B_{3u}$	n → π*	5.87	5.95	5.93	5.82
	$2\,^1B_{3g}$	π → π*	6.78	7.36	7.23	7.27
	$2\,^1B_{1u}$	π → π*	7.73	7.80	7.83	7.82
Formamide	$1\,^1A''$	n → π*	5.93	5.54	5.65	5.65

Molecule	State	Type	NEVPT2	NEVPT3	NEVPT4	CC3
	$2\,^1A'$	$\pi \rightarrow \pi^*$	7.62	8.00	7.58	8.27
	$3\,^1A'$	$\pi \rightarrow \pi^*$	10.84	11.05	10.75	10.93
Acetamide	$1\,^1A''$	$n \rightarrow \pi^*$	5.96	5.59	5.68	5.69
	$2\,^1A'$	$\pi \rightarrow \pi^*$	7.48	7.95	7.54	7.67
	$3\,^1A'$	$\pi \rightarrow \pi^*$	10.31	10.75	10.38	10.50
Propanamide	$1\,^1A''$	$n \rightarrow \pi^*$	6.05	5.64	5.73	5.72
	$2\,^1A'$	$\pi \rightarrow \pi^*$	7.41	7.91	7.49	7.62
	$3\,^1A'$	$\pi \rightarrow \pi^*$	10.16	10.62	10.23	10.06
Cytosine	$2\,^1A'$	$\pi \rightarrow \pi^*$	4.77	4.80	4.75	4.72[a]
	$1\,^1A''$	$n \rightarrow \pi^*$	5.50	5.29	5.38	5.16[a]
	$2\,^1A''$	$n \rightarrow \pi^*$	5.73	5.58	5.65	5.52[a]
	$3\,^1A'$	$\pi \rightarrow \pi^*$	5.70	5.88	5.79	5.61[a]
	$4\,^1A'$	$\pi \rightarrow \pi^*$	6.48	6.91	6.65	6.61[a]
	$5\,^1A'$	$\pi \rightarrow \pi^*$	6.80	7.20	7.01	
	$6\,^1A'$	$\pi \rightarrow \pi^*$	8.11	8.25	8.17	
Thymine	$1\,^1A''$	$n \rightarrow \pi^*$	4.94	5.01	4.97	4.98[a]
	$2\,^1A'$	$\pi \rightarrow \pi^*$	5.05	5.55	5.33	5.34[a]
	$3\,^1A'$	$\pi \rightarrow \pi^*$	6.43	6.63	6.51	6.34[a]
	$2\,^1A''$	$n \rightarrow \pi^*$	6.46	6.48	6.48	6.45[a]
	$4\,^1A'$	$\pi \rightarrow \pi^*$	6.34	7.00	6.66	6.71[a]
	$3\,^1A''$	$n \rightarrow \pi^*$	6.62	7.24	6.96	
	$4\,^1A''$	$n \rightarrow \pi^*$	7.39	7.68	7.57	
	$5\,^1A'$	$\pi \rightarrow \pi^*$	7.36	7.90	7.56	

Molecule	State	Type	NEVPT2	NEVPT3	NEVPT4	CC3
Uracil	1 $^1A''$	n → π*	4.89	4.96	4.92	4.90[a]
	2 $^1A'$	π → π*	5.29	5.64	5.48	5.44[a]
	3 $^1A'$	π → π*	6.31	6.55	6.41	6.29[a]
	2 $^1A''$	n → π*	6.39	6.41	6.41	6.32[a]
	3 $^1A''$	n → π*	6.62	7.26	6.96	6.77[a]
	4 $^1A'$	π → π*	6.61	7.12	6.84	6.84[a]
	4 $^1A''$	n → π*	7.24	7.53	7.41	7.12[a]
	5 $^1A'$	π → π*	7.40	7.84	7.54	7.93[a]
Adenine	2 $^1A'$	π → π*	5.07	5.62	5.42	5.18[a]
	3 $^1A'$	π → π*	5.43	5.23	5.30	5.39[a]
	1 $^1A''$	n → π*	5.35	5.48	5.47	5.34[a]
	2 $^1A''$	n → π*	6.06	6.12	6.12	5.96[a]
	4 $^1A'$	π → π*	6.45	7.05	6.79	6.53[a]
	5 $^1A'$	π → π*	6.82	7.03	6.93	
	6 $^1A'$	π → π*	6.95	6.83	6.85	
	7 $^1A'$	π → π*	7.72	7.85	7.79	

[a] Value is taken from Ref. [53]

Table S3 Triplet Vertical Excitation Energies ΔE (eV) (canonicalization is state averaged)

Molecule	State	Type	NEVPT2	NEVPT3	NEVPT4	CC3
Ethene	1 $^3B_{1u}$	π → π*	4.60	4.43	4.47	4.48
E-Butadiene	1 ^3B_u	π → π*	3.38	3.33	3.36	3.32
	1 ^3A_g	π → π*	5.27	5.13	5.18	5.17
all-E-Hexatriene	1 ^3B_u	π → π*	2.73	2.72	2.74	2.69

Molecule	State	Type	NEVPT2	NEVPT3	NEVPT4	CC3
	$1\ ^3A_g$	π → π*	4.39	4.31	4.35	4.32
all-E-Octatetraene	$1\ ^3B_u$	π → π*	2.32	2.34	2.36	2.30
	$1\ ^3A_g$	π → π*	3.72	3.68	3.72	3.67
Cyclopropene	$1\ ^3B_2$	π → π*	4.50	4.32	4.35	4.34
	$1\ ^3B_1$	σ → π*	6.57	6.64	6.63	6.62
Cyclopentadiene	$1\ ^3B_2$	π → π*	3.32	3.25	3.28	3.25
	$1\ ^3A_1$	π → π*	5.22	5.06	5.11	5.09
Norbornadine	$1\ ^3A_2$	π → π*	3.80	3.78	3.80	3.72
	$1\ ^3B_2$	π → π*	4.30	4.20	4.22	4.16
Benzene	$1\ ^3B_{1u}$	π → π*	4.31	4.06	4.14	4.12
	$1\ ^3E_{1u}$	π → π*	4.98	4.90	4.92	4.90
	$1\ ^3B_{2u}$	π → π*	5.47	6.19	5.97	6.04
	$1\ ^3E_{2g}$	π → π*	7.59	7.31	7.40	7.49
Naphthalene	$1\ ^3B_{2u}$	π → π*	3.26	3.12	3.18	3.11
	$1\ ^3B_{3u}$	π → π*	4.24	4.20	4.23	4.18
	$1\ ^3B_{1g}$	π → π*	4.57	4.46	4.51	4.47
	$2\ ^3B_{2u}$	π → π*	4.70	4.63	4.67	4.64
	$2\ ^3B_{3u}$	π → π*	4.44	5.28	5.05	5.11
	$1\ ^3A_g$	π → π*	5.58	5.52	5.57	5.52
	$2\ ^3B_{1g}$	π → π*	5.81	6.78	6.45	6.48
	$2\ ^3A_g$	π → π*	6.12	6.97	6.73	6.47
	$3\ ^3A_g$	π → π*	6.52	6.37	6.43	6.79
	$3\ ^3B_{1g}$	π → π*	6.78	6.61	6.68	6.76

Molecule	State	Type	NEVPT2	NEVPT3	NEVPT4	CC3
Furan	1 $^3B_2$	π → π*	4.35	4.09	4.18	4.17
	1 $^3A_1$	π → π*	5.65	5.48	5.54	5.48
Pyrrole	1 $^3B_2$	π → π*	4.73	4.43	4.52	4.48
	1 $^3A_1$	π → π*	5.68	5.53	5.59	5.51
Imidazole	1 $^3A'$	π → π*	4.73	4.69	4.68	4.69
	2 $^3A'$	π → π*	5.87	5.83	5.84	5.79
	1 $^3A''$	n → π*	6.43	6.33	6.36	6.37
	3 $^3A'$	π → π*	6.59	6.80	6.67	6.55
	4 $^3A'$	π → π*	7.08	7.37	7.23	7.42
	2 $^3A''$	n → π*	7.58	7.56	7.55	7.51
Pyridine	1 $^3A_1$	π → π*	4.47	4.18	4.28	4.25
	1 $^3B_1$	n → π*	4.58	4.55	4.55	4.50
	1 $^3B_2$	π → π*	4.93	4.94	4.95	4.86
	2 $^3A_1$	π → π*	5.13	5.08	5.12	5.05
	1 $^3A_2$	n → π*	5.46	5.60	5.52	5.46
	2 $^3B_2$	π → π*	6.43	6.99	6.82	6.40
	3 $^3B_2$	π → π*	7.22	7.16	7.18	7.83
	3 $^3A_1$	π → π*	7.83	7.49	7.60	7.66
s-Tetrazine	1 $^3B_{3u}$	n → π*	1.63	1.97	1.94	1.89
	1 ^3A_u	n → π*	3.44	3.63	3.67	3.52
	1 $^3B_{1g}$	n → π*	4.34	4.24	4.34	4.21
	1 $^3B_{1u}$	π → π*	4.55	4.16	4.31	4.33
	1 $^3B_{2u}$	π → π*	4.72	4.72	4.70	4.54

Molecule	*State*	*Type*	*NEVPT2*	*NEVPT3*	*NEVPT4*	*CC3*
	$1\ ^3B_{2g}$	$n \rightarrow \pi^*$	5.19	4.87	5.04	4.93
	$2\ ^3A_u$	$n \rightarrow \pi^*$	5.01	5.25	5.23	5.03
	$1\ ^3B_{3g}$	$n,n \rightarrow \pi^*,\pi^*$				
	$2\ ^3B_{1u}$	$\pi \rightarrow \pi^*$	5.51	5.43	5.48	5.38
	$2\ ^3B_{2g}$	$n \rightarrow \pi^*$	6.11	6.09	6.18	6.04
	$2\ ^3B_{1g}$	$n \rightarrow \pi^*$	6.55	6.47	6.57	6.60
	$2\ ^3B_{3u}$	$n \rightarrow \pi^*$	6.73	6.75	6.81	6.53
	$2\ ^3B_{2u}$	$\pi \rightarrow \pi^*$	6.41	7.47	7.09	7.36
Formaldehyde	$1\ ^3A_2$	$n \rightarrow \pi^*$	3.75	3.62	3.64	3.55
	$1\ ^3A_1$	$\pi \rightarrow \pi^*$	6.04	5.64	5.79	5.83
Acetone	$1\ ^3A_2$	$n \rightarrow \pi^*$	4.10	4.18	4.12	4.05
	$1\ ^3A_1$	$\pi \rightarrow \pi^*$	6.06	6.04	6.05	6.03
p-Benzoquinone	$1\ ^3B_{1g}$	$n \rightarrow \pi^*$	2.82	2.89	2.86	2.51
	$1\ ^3A_u$	$n \rightarrow \pi^*$	2.83	2.91	2.89	2.62
	$1\ ^3B_{1u}$	$\pi \rightarrow \pi^*$	2.92	3.01	3.07	2.96
	$1\ ^3B_{3g}$	$\pi \rightarrow \pi^*$	3.39	3.54	3.54	3.41
Formamide	$1\ ^3A''$	$n \rightarrow \pi^*$	5.54	5.26	5.38	5.36
	$1\ ^3A'$	$\pi \rightarrow \pi^*$	5.84	5.80	5.78	5.74
Acetamide	$1\ ^3A''$	$n \rightarrow \pi^*$	5.50	5.42	5.41	5.42
	$1\ ^3A'$	$\pi \rightarrow \pi^*$	5.70	5.89	5.84	5.88
Propanamide	$1\ ^3A''$	$n \rightarrow \pi^*$	5.54	5.46	5.44	5.45
	$1\ ^3A'$	$\pi \rightarrow \pi^*$	5.87	5.87	5.88	5.90

S3 Documentation of Benchmarking Results

S3.1 Unsaturated Aliphatic Hydrocarbons

S3.1.1 Ethene

Active space is (0 1 0 0 0 0 0 1)2 shown as (active orbitals) active electrons. The number of active orbitals in the parenthesis are of symmetry a_g b_{2g} b_{1g} b_{3g} a_u b_{2u} b_{1u} b_{3u}. Ground state is ^1A_g symmetry and the highest roots calculated are 1^1 A_g 1 $^1B_{1u}$ and 1 $^3B_{1u}$.

Table S4 Vertical Excitation Energies ΔE (eV) of the singlet and triplet excitations for ethylene (canonicalization is state specific)

State	*Type*	*CASSCF*	*NEVPT2*	*NEVPT3*	*NEVPT4*	*CC3*
1 $^1B_{1u}$	*π → π**	*4.28*	*8.64*	*8.74*	*8.45*	*8.37*
1 $^3B_{1u}$	*π → π**	*9.44*	*4.60*	*4.43*	*4.47*	*4.48*

S3.1.2 E-Butadiene

Active space is (0 2 2 0)4 shown as (active orbitals) active electrons. The number of active orbitals in the parenthesis are of symmetry a_g b_g a_u b_u. Ground state is ^1A_g symmetry and the highest roots calculated are 3^1A_g, 2^1B_g, 1^3A_u, 1^3B_u.

Table S5 Vertical Excitation Energies ΔE (eV) of the singlet and triplet excitations for E-butadiene (canonicalization is state specific)

State	*Type*	*CASSCF*	*NEVPT2*	*NEVPT3*	*NEVPT4*	*CC3*
1 ^1B_u	*π → π**	*8.44*	*6.21*	*6.93*	*6.57*	*6.58*
2 ^1A_g	*π → π**	*6.44*	*6.8*	*6.56*	*6.63*	*6.77*
1 ^3B_u	*π → π**	*3.34*	*3.38*	*3.33*	*3.36*	*3.32*
1 ^3A_g	*π → π**	*5.09*	*5.27*	*5.13*	*5.18*	*5.17*

S3.1.3 E-Hexatriene

Active space is (0 3 3 0)6 shown as (active orbitals) active electrons. The number of active orbitals in the parenthesis are of symmetry a_g b_g a_u b_u. Ground state is ^1A_g symmetry and the highest roots calculated are 2^1A_g, 2^1B_g, 1^3A_u, 1^3B_u.

Table S6 Vertical Excitation Energies ΔE (eV) of the singlet and triplet excitations for E-hexatriene (canonicalization is state specific)

State	*Type*	*CASSCF*	*NEVPT2*	*NEVPT3*	*NEVPT4*	*CC3*
1 ^1B_u	*π → π**	*7.49*	*4.84*	*5.94*	*5.53*	*5.58*
2 ^1A_g	*π → π**	*5.90*	*5.56*	*5.44*	*5.47*	*5.72*
1 ^3B_u	*π → π**	*2.78*	*2.73*	*2.72*	*2.74*	*2.69*
1 ^3A_g	*π → π**	*4.33*	*4.39*	*4.31*	*4.35*	*4.32*

S3.1.4 E-Octatetraene

Active space is (0 4 4 0)8 shown as (active orbitals) active electrons. The number of active orbitals in the parenthesis are of symmetry a_g b_g a_u b_u. Ground state is ^1A_g symmetry and the highest roots calculated are 5^1A_g, 3^1B_g, 1^3A_u, 1^3B_u.

Table S7 Vertical Excitation Energies ΔE (eV) of the singlet and triplet excitations for E-octatetraene (canonicalization is state specific)

State	*Type*	*CASSCF*	*NEVPT2*	*NEVPT3*	*NEVPT4*	*CC3*
$2\ ^1A_g$	π → π*	4.68	4.72	4.64	4.68	4.97
$1\ ^1B_u$	π → π*	6.92	4.04	5.3	4.87	4.94
$2\ ^1B_u$	π → π*	6.09	5.86	5.85	5.87	6.06
$3\ ^1A_g$	π → π*	8.98	5.97	7.13	6.66	6.5
$4\ ^1A_g$	π → π*	6.60	6.67	6.46	6.51	6.81
$3\ ^1B_u$	π → π*	8.43	8.35	8.2	8.25	7.91
$1\ ^3B_u$	π → π*	2.43	2.32	2.34	2.36	2.30
$1\ ^3A_g$	π → π*	3.75	3.72	3.68	3.72	3.67

S3.1.5 Cyclopropene

Active space is (0 1 1 1)4 shown as (active orbitals) active electrons. The number of active orbitals in the parenthesis are of symmetry a_1 b_2 a_2 b_1. Ground state is $^1A_1$ symmetry and the highest roots calculated are 1^1A_1, 1^1B_1, 1^1B_2, 1^3B_1, 1^3B_2.

Table S8 Vertical Excitation Energies ΔE (eV) of the singlet and triplet excitations for cyclopropane (canonicalization is state specific)

State	*Type*	*CASSCF*	*NEVPT2*	*NEVPT3*	*NEVPT4*	*CC3*
$1\,^1B_1$	σ → π*	7.27	6.84	6.95	6.91	6.90
$1\,^1B_2$	π → π*	8.59	7.03	7.40	7.14	7.10
$1\,^3B_2$	π → π*	4.18	4.50	4.32	4.35	4.34
$1\,^3B_1$	σ → π*	6.81	6.57	6.64	6.63	6.62

S3.1.6 Cyclopentadiene

Active space is (0 2 2 0)4 shown as (active orbitals) active electrons. The number of active orbitals in the parenthesis are of symmetry a_1 b_1 a_2 b_2. Ground state is $^1A_1$ symmetry and the highest roots calculated are 5^1A_1, 3^1B_2, 1^3A_1, 1^3B_2.

Table S9 Vertical Excitation Energies ΔE (eV) of the singlet and triplet excitations for cyclopentadiene (canonicalization is state specific)

State	*Type*	*CASSCF*	*NEVPT2*	*NEVPT3*	*NEVPT4*	*CC3*
$1\,^1B_2$	π → π*	7.62	5.21	6.05	5.67	5.73
$2\,^1A_1$	π → π*	6.48	6.72	6.53	6.56	6.61
$3\,^1A_1$	π → π*	10.73	8.22	9.05	8.55	8.69
$1\,^3B_2$	π → π*	3.23	3.32	3.25	3.28	3.25
$1\,^3A_1$	π → π*	5.00	5.22	5.06	5.11	5.09

S3.1.7 Norbornadiene

Active space is (1 1 1 1)4 shown as (active orbitals) active electrons. The number of active orbitals in the parenthesis are of symmetry a_1 b_2 a_2 b_1. Ground state is $^1A_1$ symmetry and the highest roots calculated are 3^1A_1, 2^1A_2, 2^1B_2, 1^3A_2, 1^3B_2.

Table S10 Vertical Excitation Energies ΔE (eV) of the singlet and triplet excitations for norbornadiene (canonicalization is state specific)

State	Type	CASSCF	NEVPT2	NEVPT3	NEVPT4	CC3
$1\ ^1A_2$	$\pi \rightarrow \pi^*$	7.64	5.04	6.02	5.64	5.64
$1\ ^1B_2$	$\pi \rightarrow \pi^*$	9.16	5.80	7.02	6.44	6.49
$2\ ^1B_2$	$\pi \rightarrow \pi^*$	10.23	6.98	8.16	7.58	7.64
$2\ ^1A_2$	$\pi \rightarrow \pi^*$	10.22	7.04	8.20	7.69	7.71
$1\ ^3A_2$	$\pi \rightarrow \pi^*$	3.83	3.80	3.78	3.80	3.72
$1\ ^3B_2$	$\pi \rightarrow \pi^*$	4.21	4.30	4.20	4.22	4.16

S3.2 Aromatic Hydrocarbons and Heterocycles

S3.2.1 Benzene

Active space is (1 1 1 0 0 1 1 1)6 shown as (active orbitals) active electrons. The number of active orbitals in the parenthesis are of symmetry a_g b_{2g} b_{1g} b_{3g} a_u b_{2u} b_{1u} b_{3u}. Ground state is ^1A_g symmetry and the highest roots calculated are 1^1A_g, 1^1B_{1g}, 2^1B_{2u}, 2^1B_{3u}, 1^3A_g 2^3B_{2u}, and 1^3B_{3u}.

Table S11 Vertical Excitation Energies ΔE (eV) of the singlet and triplet excitations for benzene (canonicalization is state specific)

State	Type	CASSCF	NEVPT2	NEVPT3	NEVPT4	CC3
$1\ ^1B_{2u}$	$\pi \rightarrow \pi^*$	5.06	5.23	5.00	5.06	5.07
$1\ ^1B_{1u}$	$\pi \rightarrow \pi^*$	7.99	6.40	6.82	6.66	6.68
$1\ ^1E_{1u}$	$\pi \rightarrow \pi^*$	9.30	7.11	7.75	7.39	7.45
$2\ ^1E_{2g}$	$\pi \rightarrow \pi^*$	8.06	8.42	8.17	8.24	8.43
$1\ ^3B_{1u}$	$\pi \rightarrow \pi^*$	3.86	4.31	4.06	4.14	4.12
$1\ ^3E_{1u}$	$\pi \rightarrow \pi^*$	5.06	4.98	4.90	4.92	4.90
$1\ ^3B_{2u}$	$\pi \rightarrow \pi^*$	7.30	5.47	6.19	5.97	6.04
$1\ ^3E_{2g}$	$\pi \rightarrow \pi^*$	7.14	7.59	7.31	7.40	7.49

S3.2.2 Naphthalene

Active space is (0 2 0 3 2 0 3 0)10 shown as (active orbitals) active electrons. The number of active orbitals in the parenthesis are of symmetry a_g b_{2g} b_{1g} b_{3g} a_u b_{2u} b_{1u} b_{3u}. Ground state is ^1A_g symmetry and the highest roots calculated are 1^1A_g, 3^1B_{1g}, 5^1B_{2u}, 4^1B_{3u}, 3^3A_g, 3^3B_{1g}, 2^3B_{2u}, and 2^3B_{3u}.

Table S12 Vertical Excitation Energies ΔE (eV) of the singlet and triplet excitations for naphthalene (canonicalization is state specific)

State	*Type*	*CASSCF*	*NEVPT2*	*NEVPT3*	*NEVPT4*	*CC3*
$1\ ^1B_{3u}$	π → π*	4.27	4.37	4.25	4.30	4.27
$1\ ^1B_{2u}$	π → π*	6.6	4.37	5.27	4.99	5.03
$2\ ^1A_g$	π → π*	5.77	6.23	5.86	5.98	5.98
$1\ ^1B_{1g}$	π → π*	6.52	6.10	6.18	6.17	6.07
$2\ ^1B_{3u}$	π → π*	8.22	5.61	6.68	6.25	6.33
$2\ ^1B_{1g}$	π → π*	8.53	6.17	7.03	6.73	6.79
$2\ ^1B_{2u}$	π → π*	8.18	6.01	6.81	6.53	6.57
$3\ ^1A_g$	π → π*	6.78	6.85	6.70	6.77	6.90
$3\ ^1B_{2u}$	π → π*	10.38	7.81	8.70	8.31	8.44
$3\ ^1B_{3u}$	π → π*	7.91	7.92	7.78	7.84	8.12
$1\ ^3B_{2u}$	π → π*	3.06	3.26	3.12	3.18	3.11
$1\ ^3B_{3u}$	π → π*	4.36	4.24	4.20	4.23	4.18
$1\ ^3B_{1g}$	π → π*	4.49	4.57	4.46	4.51	4.47
$2\ ^3B_{2u}$	π → π*	4.66	4.70	4.63	4.67	4.64
$2\ ^3B_{3u}$	π → π*	6.43	4.44	5.28	5.05	5.11
$1\ ^3A_g$	π → π*	5.64	5.59	5.52	5.57	5.52
$2\ ^3B_{1g}$	π → π*	8.11	5.80	6.78	6.45	6.48
$2\ ^3A_g$	π → π*	8.22	6.11	6.97	6.73	6.47
$3\ ^3A_g$	π → π*	6.46	6.52	6.37	6.43	6.79
$3\ ^3B_{1g}$	π → π*	6.86	6.79	6.61	6.68	6.76

S3.2.3 Furan

Active space is (0 3 2 0)6 shown as (active orbitals) active electrons. The number of active orbitals in the parenthesis are of symmetry a_1 b_1 a_2 b_2. Ground state is $^1A_1$ symmetry and the highest roots calculated are 4^1A_1, 3^1B_2, 1^3A_1, and 1^3B_2.

Table S13 Vertical Excitation Energies ΔE (eV) of the singlet and triplet excitations for furan (canonicalization is state specific)

State	Type	CASSCF	NEVPT2	NEVPT3	NEVPT4	CC3
$1\ ^1B_2$	$\pi \rightarrow \pi^*$	8.34	6.44	7.02	6.72	6.60
$2\ ^1A_1$	$\pi \rightarrow \pi^*$	6.60	6.75	6.61	6.65	6.62
$3\ ^1A_1$	$\pi \rightarrow \pi^*$	10.10	8.35	8.96	8.59	8.53
$1\ ^3B_2$	$\pi \rightarrow \pi^*$	3.90	4.35	4.09	4.18	4.17
$1\ ^3A_1$	$\pi \rightarrow \pi^*$	5.40	5.65	5.48	5.54	5.48

S3.2.4 Pyrrole

Active space is (0 3 2 0)6 shown as (active orbitals) active electrons. The number of active orbitals in the parenthesis are of symmetry a_1 b_1 a_2 b_2. Ground state is $^1A_1$ symmetry and the highest roots calculated are 3^1A_1, 3^1B_2, 1^3A_1, and 1^3B_2.

Table S14 Vertical Excitation Energies ΔE (eV) of the singlet and triplet excitations for pyrrole (canonicalization is state specific)

State	Type	CASSCF	NEVPT2	NEVPT3	NEVPT4	CC3
$2\ ^1A_1$	$\pi \rightarrow \pi^*$	6.45	6.56	6.39	6.44	6.40
$1\ ^1B_2$	$\pi \rightarrow \pi^*$	8.02	6.78	7.10	6.92	6.71
$3\ ^1A_1$	$\pi \rightarrow \pi^*$	9.52	8.19	8.53	8.29	8.17
$1\ ^3B_2$	$\pi \rightarrow \pi^*$	4.21	4.73	4.43	4.52	4.48
$1\ ^3A_1$	$\pi \rightarrow \pi^*$	5.54	5.68	5.53	5.59	5.51

S3.2.5 Imidazole

Active space is (1 5)8 shown as (active orbitals) active electrons. The number of active orbitals in the parenthesis are of symmetry a′ a′′. Ground state is ^{1}A′ symmetry and the highest roots calculated are 5^{1}A′, 2^{1}A′′, 4^{3}A′, and 2^{3}A′′.

Table S15 Vertical Excitation Energies ΔE (eV) of the singlet and triplet excitations for imidazole (canonicalization is state specific)

State	*Type*	*CASSCF*	*NEVPT2*	*NEVPT3*	*NEVPT4*	*CC3*
1 ^{1}A″	*n → π**	*7.01*	*6.97*	*6.80*	*6.83*	*6.82*
2 ^{1}A′	*π → π**	*6.85*	*6.80*	*6.71*	*6.72*	*6.58*
3 ^{1}A′	*π → π**	*8.05*	*6.85*	*7.21*	*7.03*	*7.10*
2 ^{1}A″	*n → π**	*8.38*	*8.01*	*7.97*	*7.97*	*7.93*
4 ^{1}A′	*π → π**	*9.75*	*8.39*	*8.78*	*8.48*	*8.45*
1 ^{3}A′	*π → π**	*4.77*	*4.77*	*4.67*	*4.69*	*4.69*
2 ^{3}A′	*π → π**	*5.93*	*5.89*	*5.82*	*5.85*	*5.79*
1 ^{3}A″	*n → π**	*6.49*	*6.46*	*6.33*	*6.36*	*6.37*
3 ^{3}A′	*π → π**	*7.26*	*6.61*	*6.78*	*6.69*	*6.55*
4 ^{3}A′	*π → π**	*8.03*	*7.06*	*7.33*	*7.24*	*7.42*
2 ^{3}A″	*n → π**	*7.94*	*7.57*	*7.55*	*7.55*	*7.51*

S3.2.6 Pyridine

Active space is (0 4 2 0)6 shown as (active orbitals) active electrons. The number of active orbitals in the parenthesis are of symmetry a_1 b_1 a_2 b_2. Ground state is $^1A_1$ symmetry and the highest roots calculated are 4^1A_1, 3^1B_2, 1^1A_2, 3^1B_1, 3^3A_1, 3^3B_2, 1^3A_2, and 1^3B_1. States marked by (*) have used a larger active space of (1 4 2 0)8.

Table S16 Vertical Excitation Energies ΔE (eV) of the singlet and triplet excitations for pyridine (canonicalization is state specific)

State	*Type*	*CASSCF*	*NEVPT2*	*NEVPT3*	*NEVPT4*	*CC3*
$1\ ^1B_2$	$\pi \rightarrow \pi^*$	5.07	5.33	5.10	5.18	5.15
$1\ ^1B_1$ (*)	$n \rightarrow \pi^*$	5.22	5.26	5.14	5.14	5.05
$1\ ^1A_2$ (*)	$n \rightarrow \pi^*$	5.96	5.46	5.62	5.53	5.50
$2\ ^1A_1$	$\pi \rightarrow \pi^*$	7.94	7.09	7.33	7.28	6.85
$3\ ^1A_1$	$\pi \rightarrow \pi^*$	9.58	7.23	8.05	7.62	7.70
$2\ ^1B_2$	$\pi \rightarrow \pi^*$	9.78	7.10	8.09	7.60	7.59
$4\ ^1A_1$	$\pi \rightarrow \pi^*$	8.4	8.03	8.05	8.06	8.68
$3\ ^1B_2$	$\pi \rightarrow \pi^*$	8.33	8.53	8.30	8.35	8.77
$1\ ^3A_1$	$\pi \rightarrow \pi^*$	3.94	4.47	4.18	4.28	4.25
$1\ ^3B_1$ (*)	$n \rightarrow \pi^*$	4.64	4.58	4.55	4.55	4.50
$1\ ^3B_2$	$\pi \rightarrow \pi^*$	5.08	4.94	4.93	4.95	4.86
$2\ ^3A_1$	$\pi \rightarrow \pi^*$	5.13	5.13	5.08	5.12	5.05
$1\ ^3A_2$ (*)	$n \rightarrow \pi^*$	5.96	5.46	5.60	5.52	5.46
$2\ ^3B_2$	$\pi \rightarrow \pi^*$	7.89	6.41	6.98	6.81	6.40
$3\ ^3B_2$	$\pi \rightarrow \pi^*$	7.33	7.23	7.15	7.18	7.83
$3\ ^3A_1$	$\pi \rightarrow \pi^*$	7.29	7.83	7.49	7.60	7.66

S3.2.7 Pyrazine

Active space is (1 2 1 0 1 0 1 2)10 shown as (active orbitals) active electrons. The number of active orbitals in the parenthesis are of symmetry a_g b_{2g} b_{1g} b_{3g} a_u b_{2u} b_{1u} b_{3u}. Ground state is ^1A_g symmetry and the highest roots calculated are 2^1A_g, 2^1A_u, 2^1B_{1g}, 1^1B_{2g}, 1^1B_{3g}, 2^1B_{1u}, 2^1B_{2u}, 1^1B_{3u}.

Table S17 Vertical Excitation Energies ΔE (eV) of the singlet and triplet excitations for pyrazine (canonicalization is state specific)

State	*Type*	*CASSCF*	*NEVPT2*	*NEVPT3*	*NEVPT4*	*CC3*
1 $^1B_{3u}$	*n → π**	*4.83*	*4.20*	*4.36*	*4.34*	*4.24*
1 ^1A_u	*n → π**	*6.24*	*4.93*	*5.27*	*5.19*	*5.05*
1 $^1B_{2u}$	*π → π**	*5.06*	*5.31*	*5.03*	*5.08*	*5.02*
1 $^1B_{2g}$	*n → π**	*5.80*	*5.86*	*5.68*	*5.75*	*5.74*
1 $^1B_{1g}$	*n → π**	*7.44*	*6.77*	*6.86*	*6.87*	*6.75*
1 $^1B_{1u}$	*π → π**	*8.60*	*6.76*	*7.25*	*7.04*	*7.07*
2 $^1B_{1u}$	*π → π**	*10.42*	*7.72*	*8.53*	*7.98*	*8.06*
2 $^1B_{2u}$	*π → π**	*10.02*	*7.43*	*8.31*	*7.87*	*8.05*
1 $^1B_{3g}$	*π → π**	*8.32*	*8.73*	*8.46*	*8.55*	*8.77*
2 ^1A_g	*π → π**	*8.32*	*8.87*	*8.51*	*8.61*	*8.69*

S3.2.8 Pyrimidine

Active space is (1 1 2 4)10 shown as (active orbitals) active electrons. The number of active orbitals in the parenthesis are of symmetry a_1 b_2 a_2 b_1. Ground state is $^1A_1$ symmetry and the highest roots calculated are 4^1A_1, 4^1B_2, 1^1A_2, 1^1B_1.

Table S18 Vertical Excitation Energies ΔE (eV) of the singlet and triplet excitations for pyrimidine (canonicalization is state specific)

State	*Type*	*CASSCF*	*NEVPT2*	*NEVPT3*	*NEVPT4*	*CC3*
1 $^1B_1$	*n → π**	*4.92*	*4.52*	*4.58*	*4.57*	*4.50*
1 $^1A_2$	*n → π**	*5.59*	*4.81*	*5.02*	*4.98*	*4.93*
1 $^1B_2$	*π → π**	*5.32*	*5.61*	*5.32*	*5.39*	*5.36*
2 $^1A_1$	*π → π**	*8.09*	*7.42*	*7.58*	*7.50*	*7.06*
2 $^1B_2$	*π → π**	*10.25*	*7.51*	*8.44*	*7.93*	*8.01*
3 $^1A_1$	*π → π**	*9.84*	*7.74*	*8.46*	*8.06*	*7.74*

S3.2.9 Pyridazine

Active space is (1 1 2 4)10 shown as (active orbitals) active electrons. The number of active orbitals in the parenthesis are of symmetry a_1 b_2 a_2 b_1. Ground state is $^1A_1$ symmetry and the highest roots calculated are 4^1A_1, 4^1B_2, 2^1A_2, 2^1B_1.

Table S19 Vertical Excitation Energies ΔE (eV) of the singlet and triplet excitations for pyridazine (canonicalization is state specific)

State	Type	CASSCF	NEVPT2	NEVPT3	NEVPT4	CC3
$1\,^1B_1$	$n \rightarrow \pi^*$	4.62	3.92	4.08	4.03	3.92
$1\,^1A_2$	$n \rightarrow \pi^*$	5.13	4.57	4.67	4.65	4.49
$2\,^1A_1$	$\pi \rightarrow \pi^*$	4.95	5.46	5.12	5.24	5.22
$2\,^1A_2$	$n \rightarrow \pi^*$	6.36	5.89	5.87	5.86	5.74
$2\,^1B_1$	$n \rightarrow \pi^*$	7.27	6.68	6.68	6.65	6.41
$1\,^1B_2$	$\pi \rightarrow \pi^*$	8.83	7.34	7.78	7.60	6.93
$2\,^1B_2$	$\pi \rightarrow \pi^*$	9.73	7.25	8.08	7.61	7.55
$3\,^1A_1$	$\pi \rightarrow \pi^*$	9.99	7.38	8.32	7.77	7.82

S3.2.10 s-Triazine

Active space is (2 4 2 1)12 shown as (active orbitals) active electrons. The number of active orbitals in the parenthesis are of symmetry a_1 b_2 a_2 b_1. Ground state is $^1A_1$ symmetry and the highest roots calculated are 4^1A_1, 1^1B_2, 3^1A_2, 1^1B_1.

Table S20 Vertical Excitation Energies ΔE (eV) of the singlet and triplet excitations for s-triazine (canonicalization is state specific)

State	Type	CASSCF	NEVPT2	NEVPT3	NEVPT4	CC3
$1\,^1A_1''$	$n \rightarrow \pi^*$	6.14	4.65	5.08	4.99	4.78
$1\,^1A_2''$	$n \rightarrow \pi^*$	5.13	4.88	4.85	4.90	4.76
$1\,^1E''$	$n \rightarrow \pi^*$	5.72	4.87	5.01	5.01	4.81
$1\,^1A_2'$	$\pi \rightarrow \pi^*$	5.48	5.92	5.63	5.75	5.71
$2\,^1A_1'$	$\pi \rightarrow \pi^*$	8.59	7.20	7.71	7.51	7.41
$2\,^1E''$	$n \rightarrow \pi^*$	8.44	7.98	7.89	7.93	7.80
$1\,^1E'$	$\pi \rightarrow \pi^*$	10.42	7.94	8.81	8.35	8.04
$2\,^1E'$	$\pi \rightarrow \pi^*$	8.72	9.01	8.73	8.80	9.44

S3.2.11 s-Tetrazine

Active space is (1 2 1 1 1 1 1 2)12 shown as (active orbitals) active electrons. The number of active orbitals in the parenthesis are of symmetry a_g b_{2g} b_{1g} b_{3g} a_u b_{2u} b_{1u} b_{3u}. Ground state is ^1A_g symmetry and the highest roots calculated are 5^1A_g, 3^1A_u, 4^1B_{1g}, 3^1B_{2g}, 3^1B_{3g}, 3^1B_{1u}, 5^1B_{2u}, 3^1B_{3u}, 2^3A_u, 2^3B_{1g}, 2^3B_{2g}, 1^3B_{3g}, 2^3B_{1u}, 3^3B_{2u}, 2^3B_{3u}.

Table S21 Vertical Excitation Energies ΔE (eV) of the singlet and triplet excitations for s-tetrazine (canonicalization is state specific)

State	*Type*	*CASSCF*	*NEVPT2*	*NEVPT3*	*NEVPT4*	*CC3*
1 $^1B_{3u}$	*n → π**	*3.48*	*2.41*	*2.63*	*2.61*	*2.53*
1 ^1A_u	*π → π**	*4.89*	*3.76*	*3.97*	*3.97*	*3.79*
1 $^1B_{1g}$	*n → π**	*5.62*	*5.17*	*5.04*	*5.12*	*4.97*
1 $^1B_{2u}$	*π → π**	*5.16*	*5.47*	*4.98*	*5.17*	*5.12*
1 $^1B_{2g}$	*n → π**	*5.47*	*5.53*	*5.20*	*5.35*	*5.34*
1 $^1B_{3g}$	*n,n → π*,π**	*7.04*	*6.30*	*6.37*	*6.40*	
2 ^1A_u	*π → π**	*6.43*	*5.70*	*5.69*	*5.69*	*5.46*
2 $^1B_{2g}$	*n → π**	*7.01*	*6.28*	*6.30*	*6.36*	*6.23*
2 $^1B_{1g}$	*n → π**	*7.30*	*6.83*	*6.70*	*6.82*	*6.87*
3 $^1B_{1g}$	*n → π**	*7.76*	*7.02*	*6.94*	*7.04*	*7.08*
2 $^1B_{3u}$	*n → π**	*7.89*	*7.11*	*7.03*	*7.06*	*6.67*
1 $^1B_{1u}$	*π → π**	*9.54*	*6.76*	*7.56*	*7.31*	*7.45*
2 $^1B_{1u}$	*π → π**	*10.39*	*6.87*	*7.97*	*7.40*	*7.79*
2 $^1B_{2u}$	*π → π**	*8.86*	*8.33*	*8.16*	*8.22*	*8.51*
2 $^1B_{3g}$	*π → π**	*9.72*	*8.10*	*8.51*	*8.37*	*8.47*
1 $^3B_{3u}$	*n → π**	*2.83*	*1.64*	*1.97*	*1.94*	*1.89*
1 ^3A_u	*n → π**	*4.52*	*3.42*	*3.63*	*3.66*	*3.52*
1 $^3B_{1g}$	*n → π**	*4.79*	*4.33*	*4.24*	*4.34*	*4.21*
1 $^3B_{1u}$	*π → π**	*3.79*	*4.55*	*4.16*	*4.31*	*4.33*
1 $^3B_{2u}$	*π → π**	*4.92*	*4.72*	*4.53*	*4.63*	*4.54*
1 $^3B_{2g}$	*n → π**	*5.15*	*5.19*	*4.87*	*5.05*	*4.93*
2 ^3A_u	*n → π**	*6.16*	*5.03*	*5.24*	*5.24*	*5.03*
1 $^3B_{3g}$	*n,n → π*,π**	*7.05*	*7.81*	*7.39*	*7.56*	
2 $^3B_{1u}$	*π → π**	*5.39*	*5.51*	*5.43*	*5.48*	*5.38*
2 $^3B_{2g}$	*n → π**	*6.84*	*6.11*	*6.09*	*6.18*	*6.04*
2 $^3B_{1g}$	*n → π**	*7.08*	*6.55*	*6.47*	*6.57*	*6.60*
2 $^3B_{3u}$	*n → π**	*7.54*	*6.72*	*6.75*	*6.81*	*6.53*
2 $^3B_{2u}$	*π → π**	*8.99*	*6.39*	*7.28*	*7.05*	*7.36*

S3.3 Aldehydes, Ketones and Amides

S3.3.1 Formaldehyde

Active space is (0 2 0 1)4 shown as (active orbitals) active electrons. The number of active orbitals in the parenthesis are of symmetry a_1 b_1 a_2 b_2. Ground state is $^1A_1$ symmetry and the highest roots calculated are 4^1A_1, 1^1A_2, 1^1B_1, 1^3A_1, 1^3A_2. States marked by (*) have used a different active space to include σ orbitals. This active space is (1 2 0 0)4.

Table S22 Vertical Excitation Energies ΔE (eV) of the singlet and triplet excitations for formaldehyde (canonicalization is state specific)

State	*Type*	*CASSCF*	*NEVPT2*	*NEVPT3*	*NEVPT4*	*CC3*
$1\,^1A_2$	n → π*	3.95	4.22	4.00	4.02	3.95
$1\,^1B_1$ (*)	σ → π*	11.82	9.40	9.21	9.23	9.18
$2\,^1A_1$	π → π*	12.39	8.79	10.18	9.20	9.53
$1\,^3A_2$	n → π*	3.61	3.75	3.62	3.64	3.55
$1\,^3A_1$	π → π*	5.27	6.04	5.64	5.79	5.83

S3.3.2 Acetone

Active space is (2 1 0 2)6 shown as (active orbitals) active electrons. The number of active orbitals in the parenthesis are of symmetry a_1 b_2 a_2 b_1. Ground state is $^1A_1$ symmetry and the highest roots calculated are 4^1A_1, 1^1A_2, 1^1B_1, 1^3A_1, 1^3A_2.

Table S23 Vertical Excitation Energies ΔE (eV) of the singlet and triplet excitations for acetone (canonicalization is state specific)

State	*Type*	*CASSCF*	*NEVPT2*	*NEVPT3*	*NEVPT4*	*CC3*
$1\,^1A_2$	n → π*	4.81	4.47	4.54	4.47	4.40
$1\,^1B_1$	σ → π*	10.32	9.50	9.55	9.42	9.17
$2\,^1A_1$	π → π*	10.79	9.28	9.48	9.17	9.65
$1\,^3A_2$	n → π*	4.49	4.10	4.18	4.12	4.05
$1\,^3A_1$	π → π*	6.14	6.06	6.04	6.05	6.03

S3.3.3 p-Benzoquinone

Active space is (0 3 1 1 1 1 0 3)12 shown as (active orbitals) active electrons. The number of active orbitals in the parenthesis are of symmetry a_g b_{2g} b_{1g} b_{3g} a_u b_{2u} b_{1u} b_{3u}. Ground state is ^1A_g symmetry and the highest roots calculated are 4^1A_u, 4^1B_{1g}, 3^1B_{2g}, 3^1B_{3g}, 4^1B_{1u}, 2^1B_{2u}, 3^1B_{3u}, 2^3A_u, 2^3B_{1g}, 2^3B_{3g}, 2^3B_{1u}.

Table S24 Vertical Excitation Energies ΔE (eV) of the singlet and triplet excitations for p-benzoquinone (canonicalization is state specific)

State	Type	CASSCF	NEVPT2	NEVPT3	NEVPT4	CC3
$1\ ^1A_u$	$n \rightarrow \pi^*$	3.47	2.99	3.08	3.05	2.85
$1\ ^1B_{1g}$	$n \rightarrow \pi^*$	3.48	3.00	3.07	3.03	2.75
$1\ ^1B_{3g}$	$\pi \rightarrow \pi^*$	6.06	4.35	4.97	4.83	4.59
$1\ ^1B_{1u}$	$\pi \rightarrow \pi^*$	7.97	4.85	6.01	5.61	5.62
$1\ ^1B_{3u}$	$n \rightarrow \pi^*$	6.45	5.88	5.95	5.94	5.82
$2\ ^1B_{3g}$	$\pi \rightarrow \pi^*$	8.54	6.70	7.35	7.22	7.27
$2\ ^1B_{1u}$	$\pi \rightarrow \pi^*$	8.30	7.72	7.80	7.83	7.82
$1\ ^3B_{1g}$	$n \rightarrow \pi^*$	3.27	2.82	2.89	2.86	2.51
$1\ ^3A_u$	$n \rightarrow \pi^*$	3.27	2.82	2.91	2.89	2.62
$1\ ^3B_{1u}$	$\pi \rightarrow \pi^*$	3.14	2.93	3.01	3.07	2.96
$1\ ^3B_{3g}$	$\pi \rightarrow \pi^*$	3.88	3.40	3.54	3.54	3.41

S3.3.4 Formamide

Active space is (1 3)6 shown as (active orbitals) active electrons. The number of active orbitals in the parenthesis are of symmetry a′ a′′. Ground state is $^1A'$ symmetry and the highest roots calculated are $3^1A'$, $1^1A''$, $2^3A'$, and $2^3A''$. States marked by (*) have used a larger active space of (1 4)6.

Table S25 Vertical Excitation Energies ΔE (eV) of the singlet and triplet excitations for formamide (canonicalization is state specific)

State	Type	CASSCF	NEVPT2	NEVPT3	NEVPT4	CC3
$1\ ^1A''$	$n \rightarrow \pi^*$	5.38	5.93	5.54	5.65	5.65
$2\ ^1A'$	$\pi \rightarrow \pi^*$	8.76	7.58	8.01	7.57	8.27
$3\ ^1A'$	$\pi \rightarrow \pi^*$	11.8	10.75	11.07	10.74	10.93
$1\ ^3A''$ (*)	$n \rightarrow \pi^*$	5.45	5.64	5.27	5.40	5.36
$1\ ^3A'$ (*)	$\pi \rightarrow \pi^*$	6.04	5.81	5.79	5.78	5.74

S3.3.5 Acetamide

Active space is (1 3)6 shown as (active orbitals) active electrons. The number of active orbitals in the parenthesis are of symmetry a′ a′′. Ground state is $^1A'$ symmetry and the highest roots calculated are $3^1A'$, $1^1A''$, $2^3A'$, and $2^3A''$.

Table S26 Vertical Excitation Energies ΔE (eV) of the singlet and triplet excitations for acetamide (canonicalization is state specific)

State	*Type*	*CASSCF*	*NEVPT2*	*NEVPT3*	*NEVPT4*	*CC3*
1 ^{1}A″	*n → π**	*5.47*	*5.97*	*5.60*	*5.69*	*5.69*
2 ^{1}A′	*π → π**	*8.72*	*7.48*	*7.96*	*7.53*	*7.67*
3 ^{1}A′	*π → π**	*11.71*	*10.28*	*10.76*	*10.38*	*10.50*
1 ^{3}A″	*n → π**	*5.65*	*5.50*	*5.38*	*5.41*	*5.42*
1 ^{3}A′	*π → π**	*6.26*	*5.67*	*5.89*	*5.84*	*5.88*

S3.3.6 Propanamide

Active space is (1 3)6 shown as (active orbitals) active electrons. The number of active orbitals in the parenthesis are of symmetry a′ a″. Ground state is ^{1}A′ symmetry and the highest roots calculated are 3^{1}A′, 1^{1}A″, 2^{3}A′, and 2^{3}A″. States marked by (*) have used a larger active space of (1 4)6.

Table S27 Vertical Excitation Energies ΔE (eV) of the singlet and triplet excitations for propanamide (canonicalization is state specific)

State	*Type*	*CASSCF*	*NEVPT2*	*NEVPT3*	*NEVPT4*	*CC3*
1 ^{1}A″	*n → π**	*5.49*	*5.98*	*5.62*	*5.73*	*5.72*
2 ^{1}A′	*π → π**	*8.69*	*7.42*	*7.93*	*7.48*	*7.62*
3 ^{1}A′	*π → π**	*11.64*	*10.14*	*10.64*	*10.23*	*10.06*
1 ^{3}A″	*n → π**	*5.69*	*5.55*	*5.42*	*5.45*	*5.45*
1 ^{3}A′ (*)	*π → π**	*6.23*	*5.82*	*5.86*	*5.88*	*5.90*

S3.4 Nucelobases

S3.4.1 Cytosine

Active space is (0 8)10 shown as (active orbitals) active electrons. The number of active orbitals in the parenthesis are of symmetry a′ a′′. Ground state is $^1A'$ symmetry and the highest roots calculated are $6^1A'$, $2^1A''$. States marked by (*) have used a larger active space of (2 8)14.

Table S28 Vertical Excitation Energies ΔE (eV) of the singlet and triplet excitations for Cytosine (canonicalization is state specific)

State	*Type*	*CASSCF*	*NEVPT2*	*NEVPT3*	*NEVPT4*	*CC3*
2 $^1A'$	*π → π**	*5.07*	*4.70*	*4.80*	*4.74*	*4.72*[a]
1 $^1A''$ (*)	*n → π**	*5.52*	*5.50*	*5.29*	*5.38*	*5.16*[a]
2 $^1A''$ (*)	*n → π**	*5.81*	*5.73*	*5.57*	*5.65*	*5.52*[a]
3 $^1A'$	*π → π**	*6.31*	*5.65*	*5.87*	*5.78*	*5.61*[a]
4 $^1A'$	*π → π**	*7.86*	*6.47*	*6.91*	*6.66*	*6.61*[a]
5 $^1A'$	*π → π**	*8.00*	*6.83*	*7.17*	*7.02*	
6 $^1A'$	*π → π**	*8.86*	*8.06*	*8.26*	*8.16*	

S3.4.2 Thymine

Active space is (0 9)12 shown as (active orbitals) active electrons. The number of active orbitals in the parenthesis are of symmetry a′ a′′. Ground state is $^1A'$ symmetry and the highest roots calculated are $6^1A'$, $4^1A''$. States marked by (*) have used a larger active space of (2 9)16.

Table S29 Vertical Excitation Energies ΔE (eV) of the singlet and triplet excitations for Thymine (canonicalization is state specific)

State	*Type*	*CASSCF*	*NEVPT2*	*NEVPT3*	*NEVPT4*	*CC3*
1 $^1A''$ (*)	*n → π**	*5.44*	*4.96*	*4.97*	*4.98*	*4.98*[a]
2 $^1A'$	*π → π**	*6.42*	*5.05*	*5.55*	*5.33*	*5.34*[a]
3 $^1A'$	*π → π**	*8.07*	*6.34*	*7.00*	*6.66*	*6.34*[a]
2 $^1A''$ (*)	*n → π**	*6.96*	*6.49*	*6.47*	*6.49*	*6.45*[a]
4 $^1A'$	*π → π**	*7.16*	*6.43*	*6.63*	*6.51*	*6.71*[a]
3 $^1A''$ (*)	*n → π**	*8.43*	*6.69*	*7.16*	*7.00*	
4 $^1A''$ (*)	*n → π**	*8.51*	*7.41*	*7.64*	*7.59*	
5 $^1A'$	*π → π**	*8.62*	*7.36*	*7.90*	*7.56*	

S3.4.3 Uracil

Active space is (0 8)10 shown as (active orbitals) active electrons. The number of active orbitals in the parenthesis are of symmetry a′ a′′. Ground state is $^1A'$ symmetry and the highest roots calculated are $5^1A'$, $4^1A''$. States marked by (*) have used a larger active space of (2 8)14.

Table S30 Vertical Excitation Energies ΔE (eV) of the singlet and triplet excitations for Uracil (canonicalization is state specific)

State	*Type*	*CASSCF*	*NEVPT2*	*NEVPT3*	*NEVPT4*	*CC3*
1 $^1A''$ (*)	*n → π**	*5.40*	*4.92*	*4.92*	*4.94*	*4.90[a]*
2 $^1A'$	*π → π**	*6.37*	*5.27*	*5.63*	*5.48*	*5.44[a]*
3 $^1A'$	*π → π**	*7.10*	*6.22*	*6.54*	*6.40*	*6.29[a]*
2 $^1A''$ (*)	*n → π**	*6.91*	*6.42*	*6.40*	*6.42*	*6.32[a]*
3 $^1A''$ (*)	*n → π**	*8.46*	*6.70*	*7.17*	*7.01*	*6.77[a]*
4 $^1A'$	*π → π**	*8.08*	*6.68*	*7.09*	*6.86*	*6.84[a]*
4 $^1A''$ (*)	*n → π**	*8.40*	*7.27*	*7.49*	*7.43*	*7.12[a]*
5 $^1A'$	*π → π**	*8.84*	*7.38*	*7.83*	*7.54*	*7.93[a]*

S3.4.3 Adenine

Active space is (0 10)12 shown as (active orbitals) active electrons. The number of active orbitals in the parenthesis are of symmetry a′ a′′. Ground state is $^1A'$ symmetry and the highest roots calculated are $7^1A'$, $4^1A''$. States marked by (*) have used a larger active space of (3 10)18.

Table S31 Vertical Excitation Energies ΔE (eV) of the singlet and triplet excitations for Adenine (canonicalization is state specific)

State	*Type*	*CASSCF*	*NEVPT2*	*NEVPT3*	*NEVPT4*	*CC3*
2 $^1A'$	*π → π**	*6.48*	*5.08*	*5.61*	*5.42*	*5.18[a]*
3 $^1A'$	*π → π**	*5.25*	*5.42*	*5.23*	*5.30*	*5.39[a]*
1 $^1A''$ (*)	*n → π**	*6.12*	*5.35*	*5.47*	*5.47*	*5.34[a]*
2 $^1A''$ (*)	*n → π**	*6.66*	*6.07*	*6.12*	*6.13*	*5.96[a]*
4 $^1A'$	*π → π**	*8.01*	*6.48*	*7.04*	*6.80*	*6.53[a]*
5 $^1A'$	*π → π**	*7.57*	*6.82*	*7.03*	*6.93*	
6 $^1A'$	*π → π**	*7.06*	*6.92*	*6.83*	*6.84*	
7 $^1A'$	*π → π**	*8.34*	*7.69*	*7.85*	*7.79*	

S4 Documentation of Geometric Coordinates

All coordinates are shown in Angstroms.

S4.1 Unsaturated Aliphatic Hydrocarbons

S4.1.1 Ethene

H	*0.000000*	*0.923274*	*1.238289*
H	*0.000000*	*-0.923274*	*1.238289*
H	*0.000000*	*0.923274*	*-1.238289*
H	*0.000000*	*-0.923274*	*-1.238289*
C	*0.000000*	*0.000000*	*0.668188*
C	*0.000000*	*0.000000*	*-0.668188*

S4.1.2 E-Butadiene

H	*1.080977*	*-2.558832*	*0.000000*
H	*-1.080977*	*2.558832*	*0.000000*
H	*2.103773*	*-1.017723*	*0.000000*
H	*-2.103773*	*1.017723*	*0.000000*
H	*-0.973565*	*-1.219040*	*0.000000*
H	*0.973565*	*1.219040*	*0.000000*
C	*0.000000*	*0.728881*	*0.000000*
C	*0.000000*	*-0.728881*	*0.000000*
C	*1.117962*	*-1.474815*	*0.000000*
C	*-1.117962*	*1.474815*	*0.000000*

S4.1.3 E-Hexatriene

H	*-0.953777*	*1.207691*	*0.000000*
H	*0.953777*	*-1.207691*	*0.000000*
H	*2.155816*	*0.952317*	*0.000000*
H	*-2.155816*	*-0.952317*	*0.000000*
H	*2.125769*	*3.402692*	*0.000000*
H	*-2.125769*	*-3.402692*	*0.000000*
H	*0.275642*	*3.397162*	*0.000000*
H	*-0.275642*	*-3.397162*	*0.000000*
C	*0.000000*	*0.676808*	*0.000000*
C	*0.000000*	*-0.676808*	*0.000000*
C	*1.204938*	*1.485654*	*0.000000*
C	*-1.204938*	*-1.485654*	*0.000000*
C	*1.203567*	*2.831663*	*0.000000*
C	*-1.203567*	*-2.831663*	*0.000000*

S4.1.4 E-Octatetraene

H	*0.971323*	*1.220135*	*0.000000*
H	*-0.971323*	*-1.220135*	*0.000000*
H	*-2.098079*	*0.984714*	*0.000000*
H	*2.098079*	*-0.984714*	*0.000000*
H	*-0.146883*	*3.418487*	*0.000000*
H	*0.146883*	*-3.418487*	*0.000000*
H	*-2.193461*	*4.766061*	*0.000000*
H	*2.193461*	*-4.766061*	*0.000000*
H	*-3.225681*	*3.230484*	*0.000000*
H	*3.225681*	*-3.230484*	*0.000000*
C	*0.000000*	*0.721494*	*0.000000*
C	*0.000000*	*-0.721494*	*0.000000*
C	*1.125014*	*-1.479515*	*0.000000*
C	*-1.125014*	*1.479515*	*0.000000*
C	*1.121071*	*-2.928797*	*0.000000*
C	*-1.121071*	*2.928797*	*0.000000*
C	*2.237376*	*-3.682263*	*0.000000*
C	*-2.237376*	*3.682263*	*0.000000*

S4.1.5 Cyclopropene

H	*0.912650*	*0.000000*	*1.457504*
H	*-0.912650*	*0.000000*	*1.457504*
H	*0.000000*	*-1.585659*	*-1.038624*
H	*0.000000*	*1.585659*	*-1.038624*
C	*0.000000*	*0.000000*	*0.859492*
C	*0.000000*	*-0.651229*	*-0.499559*
C	*0.000000*	*0.651229*	*-0.499559*

S4.1.6 Cyclopentadiene

H	*-0.879859*	*0.000000*	*1.874608*
H	*0.879859*	*0.000000*	*1.874608*
H	*0.000000*	*2.211693*	*0.612518*
H	*0.000000*	*-2.211693*	*0.612518*
H	*0.000000*	*1.349811*	*-1.886050*
H	*0.000000*	*-1.349811*	*-1.886050*
C	*0.000000*	*0.000000*	*1.215652*
C	*0.000000*	*-1.177731*	*0.285415*
C	*0.000000*	*1.177731*	*0.285415*
C	*0.000000*	*-0.732372*	*-0.993420*
C	*0.000000*	*0.732372*	*-0.993420*

S4.1.7 Norbornadiene

H	0.901419	0.000000	1.967823
H	-0.901419	0.000000	1.967823
H	0.000000	2.156504	0.616597
H	0.000000	-2.156504	0.616597
H	1.924341	1.340999	-1.022814
H	-1.924341	1.340999	-1.022814
H	-1.924341	-1.340999	-1.022814
H	1.924341	-1.340999	-1.022814
C	0.000000	0.000000	1.346369
C	0.000000	1.119526	0.272221
C	0.000000	-1.119526	0.272221
C	1.235500	0.672374	-0.517602
C	-1.235500	0.672374	-0.517602
C	-1.235500	-0.672374	-0.517602
C	1.235500	-0.672374	-0.517602

S4.2 Aromatic Hydrocarbons and Heterocycles

S4.2.1 Benzene

H	0.000000	2.484212	0.000000
H	2.151390	1.242106	0.000000
H	2.151390	-1.242106	0.000000
H	0.000000	-2.484212	0.000000
H	-2.151390	-1.242106	0.000000
H	-2.151390	1.242106	0.000000
C	0.000000	1.396792	0.000000
C	1.209657	0.698396	0.000000
C	1.209657	-0.698396	0.000000
C	0.000000	-1.396792	0.000000
C	-1.209657	-0.698396	0.000000
C	-1.209657	0.698396	0.000000

S4.2.2 Naphthalene

H	1.240557	2.492735	0.000000
H	1.240557	-2.492735	0.000000
H	-1.240557	2.492735	0.000000
H	-1.240557	-2.492735	0.000000
H	3.377213	1.246082	0.000000
H	3.377213	-1.246082	0.000000
H	-3.377213	1.246082	0.000000
H	-3.377213	-1.246082	0.000000
C	0.000000	0.716253	0.000000
C	0.000000	-0.716253	0.000000
C	1.241539	1.403577	0.000000
C	1.241539	-1.403577	0.000000
C	-1.241539	1.403577	0.000000
C	-1.241539	-1.403577	0.000000
C	2.432418	0.707325	0.000000
C	2.432418	-0.707325	0.000000
C	-2.432418	0.707325	0.000000
C	-2.432418	-0.707325	0.000000

S4.2.3 Furan

H	*0.000000*	*2.051058*	*0.851533*
H	*0.000000*	*-2.051058*	*0.851533*
H	*0.000000*	*1.371979*	*-1.821224*
H	*0.000000*	*-1.371979*	*-1.821224*
C	*0.000000*	*1.095840*	*0.348301*
C	*0.000000*	*-1.095840*	*0.348301*
C	*0.000000*	*0.714027*	*-0.963274*
C	*0.000000*	*-0.714027*	*-0.963274*
O	*0.000000*	*0.000000*	*1.164881*

S4.2.4 Pyrrole

H	*0.000000*	*2.114611*	*0.770889*
H	*0.000000*	*-2.114611*	*0.770889*
H	*0.000000*	*1.358585*	*-1.850224*
H	*0.000000*	*-1.358585*	*-1.850224*
H	*0.000000*	*0.000000*	*2.130670*
C	*0.000000*	*1.125828*	*0.333870*
C	*0.000000*	*-1.125828*	*0.333870*
C	*0.000000*	*0.709235*	*-0.984789*
C	*0.000000*	*-0.709235*	*-0.984789*
N	*0.000000*	*0.000000*	*1.119862*

S4.2.5 Imidazole

H	*0.000000*	*2.119822*	*0.714354*
H	*0.000000*	*1.202262*	*-1.904898*
H	*0.000000*	*-2.104815*	*0.663782*
H	*0.000000*	*-0.010302*	*2.116597*
C	*0.000000*	*1.120107*	*0.305897*
C	*0.000000*	*0.635508*	*-0.983749*
C	*0.000000*	*-1.091835*	*0.283881*
N	*0.000000*	*-0.741378*	*-0.994001*
N	*0.000000*	*0.000000*	*1.104571*

S4.2.6 Pyridine

H	*0.000000*	*2.061947*	*1.308539*
H	*0.000000*	*-2.061947*	*1.308539*
H	*0.000000*	*2.156804*	*-1.184054*
H	*0.000000*	*-2.156804*	*-1.184054*
H	*0.000000*	*0.000000*	*-2.475074*
C	*0.000000*	*1.145417*	*0.721005*
C	*0.000000*	*-1.145417*	*0.721005*
C	*0.000000*	*1.197637*	*-0.673735*
C	*0.000000*	*-1.197637*	*-0.673735*
C	*0.000000*	*0.000000*	*-1.387901*
N	*0.000000*	*0.000000*	*1.426610*

S4.2.7 Pyrazine

H	0.000000	2.068464	1.258236
H	0.000000	-2.068464	1.258236
H	0.000000	-2.068464	-1.258236
H	0.000000	2.068464	-1.258236
C	0.000000	1.135920	0.697884
C	0.000000	-1.135920	0.697884
C	0.000000	-1.135920	-0.697884
C	0.000000	1.135920	-0.697884
N	0.000000	0.000000	1.417402
N	0.000000	0.000000	-1.417402

S4.2.8 Pyrimidine

H	0.000000	2.156577	1.120194
H	0.000000	0.000000	-2.400372
H	0.000000	0.000000	2.440390
H	0.000000	-2.156577	1.120194
C	0.000000	1.186677	0.626210
C	0.000000	0.000000	-1.312618
C	0.000000	0.000000	1.354942
C	0.000000	-1.186677	0.626210
N	0.000000	1.203516	-0.717777
N	0.000000	-1.203516	-0.717777

S4.2.9 Pyridazine

H	0.000000	2.409473	-0.149324
H	0.000000	-2.409473	-0.149324
H	0.000000	1.271227	2.102636
H	0.000000	-1.271227	2.102636
C	0.000000	1.325691	-0.063084
C	0.000000	-1.325691	-0.063084
C	0.000000	0.693091	1.182942
C	0.000000	-0.693091	1.182942
N	0.000000	0.674207	-1.238923
N	0.000000	-0.674207	-1.238923

S4.2.10 s-Triazine

H	0.000000	0.000000	2.386070
H	0.000000	-2.066397	-1.193035
H	0.000000	2.066397	-1.193035
C	0.000000	0.000000	1.298338
C	0.000000	1.124394	-0.649169
C	0.000000	-1.124394	-0.649169
N	0.000000	0.000000	-1.379443
N	0.000000	1.194633	0.689722
N	0.000000	-1.194633	0.689722

S4.2.11 s-Tetrazine

H	0.000000	0.000000	-2.354782
H	0.000000	0.000000	2.354782
C	0.000000	0.000000	1.269037
C	0.000000	0.000000	-1.269037
N	0.000000	1.204566	0.670425
N	0.000000	1.204566	-0.670425
N	0.000000	-1.204566	0.670425
N	0.000000	-1.204566	-0.670425

S4.3 Aldehydes, Ketones and Amides

S4.3.1 Formaldehyde

H	0.000000	0.934473	-0.588078
H	0.000000	-0.934473	-0.588078
C	0.000000	0.000000	0.000000
O	0.000000	0.000000	1.221104

S4.3.2 Acetone

H	0.000000	2.136732	-0.112445
H	0.000000	-2.136732	-0.112445
H	-0.881334	1.333733	-1.443842
H	-0.881334	-1.333733	-1.443842
H	0.881334	1.333733	-1.443842
H	0.881334	-1.333733	-1.443842
C	0.000000	0.000000	0.000000
C	0.000000	1.287253	-0.795902
C	0.000000	-1.287253	-0.795902
O	0.000000	0.000000	1.227600

S4.3.3 p-Benzoquinone

H	0.000000	2.182962	1.259280
H	0.000000	-2.182962	1.259280
H	0.000000	-2.182962	-1.259280
H	0.000000	2.182962	-1.259280
C	0.000000	0.000000	1.441072
C	0.000000	0.000000	-1.441072
C	0.000000	1.266637	0.674578
C	0.000000	-1.266637	0.674578
C	0.000000	-1.266637	-0.674578
C	0.000000	1.266637	-0.674578
O	0.000000	0.000000	2.678504
O	0.000000	0.000000	-2.678504

S4.3.4 Formamide

H	-0.927427	-0.600301	0.000000
H	1.070498	-1.782390	0.000000
H	2.024514	-0.325050	0.000000
C	0.000000	0.000000	0.000000
O	0.000000	1.225060	0.000000
N	1.119392	-0.775069	0.000000

S4.3.5 Acetamide

H	1.173203	-1.735754	0.000000
H	2.035830	-0.226200	0.000000
H	-2.121178	-0.156088	0.000000
H	-1.310640	-1.472734	0.885499
H	-1.310640	-1.472734	-0.885499
C	0.000000	0.000000	0.000000
C	-1.267035	-0.831605	0.000000
O	0.000000	1.229432	0.000000
N	1.158961	-0.727714	0.000000

S4.3.6 Propanamide

H	1.171881	-1.734644	0.000000
H	2.036497	-0.225525	0.000000
H	-1.256730	-1.492360	0.877192
H	-1.256730	-1.492360	-0.877192
H	-3.420921	-0.590418	0.000000
H	-2.544300	0.678537	-0.880205
H	-2.544300	0.678537	0.880205
C	0.000000	0.000000	0.000000
C	-1.272720	-0.833211	0.000000
C	-2.523363	0.033790	0.000000
O	0.000000	1.230366	0.000000
N	1.159094	-0.726405	0.000000

S4.4 Nucelobases

S4.4.1 Cytosine

H	-2.114860	-1.429678	0.000000
H	-0.173973	-2.806186	0.000000
H	2.073228	-1.658021	0.000000
H	3.175240	0.564335	0.000000
H	2.235202	2.033636	0.000000
C	-0.060783	-1.726152	0.000000
C	1.144884	-1.099470	0.000000
C	1.107049	0.338190	0.000000
C	-1.227573	0.430359	0.000000
O	-2.315109	0.998271	0.000000
N	0.000000	1.058130	0.000000
N	-1.201178	-0.989148	0.000000
N	2.278974	1.024187	0.000000

S4.4.2 Thymine

H	0.217481	-2.676720	0.000000
H	2.052694	0.924773	0.000000
H	-1.943101	-1.709021	0.000000
H	-3.360610	0.309754	0.000000
H	-2.616463	1.665008	0.879105
H	-2.616463	1.665008	-0.879105
C	1.356951	-0.994496	0.000000
C	0.000000	1.121102	0.000000
C	-1.214538	0.306431	0.000000
C	-1.085764	-1.041812	0.000000
C	-2.529824	1.020445	0.000000
O	2.444132	-1.558490	0.000000
O	0.023681	2.350992	0.000000
N	0.145112	-1.666249	0.000000
N	1.192460	0.382130	0.000000

S4.4.3 Uracil

H	-2.025413	-1.517742	0.000000
H	-0.021861	1.995767	0.000000
H	2.182391	-1.602586	0.000000
H	-0.026659	-2.791719	0.000000
C	-1.239290	0.359825	0.000000
C	1.279718	0.392094	0.000000
C	1.243729	-1.064577	0.000000
C	0.055755	-1.709579	0.000000
O	-2.308803	0.954763	0.000000
O	2.287387	1.092936	0.000000
N	-1.139515	-1.026364	0.000000
N	0.000000	0.978951	0.000000

S4.4.3 Adenine

H	0.974930	-3.075149	0.000000
H	2.134658	2.075802	0.000000
H	3.312010	0.776987	0.000000
H	-3.052077	-0.334232	0.000000
H	-2.711876	2.203052	0.000000
C	0.662834	-2.032900	0.000000
C	1.359313	0.172553	0.000000
C	0.000000	0.547434	0.000000
C	-0.924835	-0.500714	0.000000
C	-1.906806	1.478795	0.000000
N	-0.658577	-1.817838	0.000000
N	1.672594	-1.133202	0.000000
N	-2.150759	0.128726	0.000000
N	-0.616118	1.783396	0.000000
N	2.352763	1.090709	0.000000
